# Supramolecular Ionic Liquid Gels for Enzyme Entrapment

**DOI:** 10.1021/acssuschemeng.3c00517

**Published:** 2023-04-24

**Authors:** Hasan T. Imam, Kyle Hill, Andrew Reid, Stefan Mix, Patricia C. Marr, Andrew C. Marr

**Affiliations:** † School of Chemistry and Chemical Engineering, Queen’s University Belfast, UK, David Keir Building, Stranmillis Road, Belfast, Northern Ireland, United Kingdom BT9 5AG; ‡ Department of Biocatalysis, Almac Bioscience, Almac Group, Almac House, 20 Seagoe Industrial Estate, Craigavon, Belfast, Northern Ireland, United Kingdom BT63 5QD

**Keywords:** Enzyme entrapment, Ionic liquid, Supramolecular gel, Ionic liquid gel, Low molecular weight gelators

## Abstract

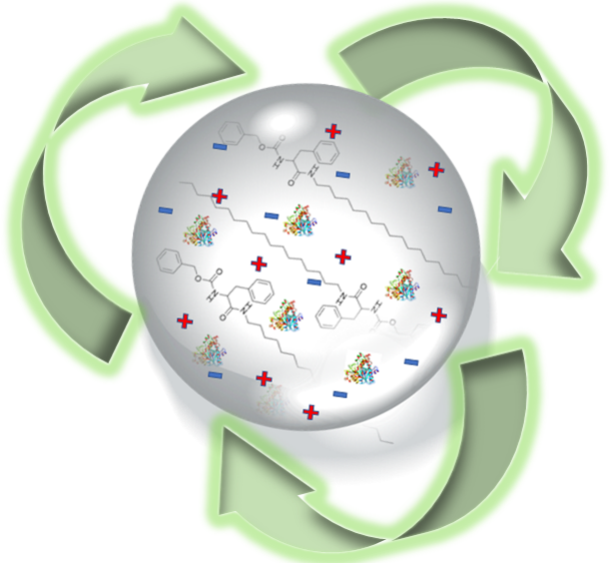

Reported herein is an entrapment method for enzyme immobilization that does not require the formation of new covalent bonds. Ionic liquid supramolecular gels are formed containing enzymes that can be shaped into gel beads and act as recyclable immobilized biocatalysts. The gel was formed from two components, a hydrophobic phosphonium ionic liquid and a low molecular weight gelator derived from the amino acid phenylalanine. Gel-entrapped lipase from *Aneurinibacillus thermoaerophilus* was recycled for 10 runs over 3 days without loss of activity and retained activity for at least 150 days. The procedure does not form covalent bonds upon gel formation, which is supramolecular, and no bonds are formed between the enzyme and the solid support.

## Abstract 2

Reported is an entrapment method for lipase immobilization in a supramolecular ionic liquid gel that does not require the formation of new covalent bonds.

## Introduction

The drive to improve the sustainability of chemical processes is leading to an increased adoption of biocatalysts, particularly in fine chemicals and pharmaceuticals.^1−6^ In these industries, selectivity is key, and the main competitors to isolated enzyme biocatalysts are homogeneous catalysts comprising metal complexes or organocatalysts. The expansion of biocatalysis is being supported by improved techniques of synthetic and molecular biology and driven by an understanding of genetics and protein structure that enables the systematic manipulation of protein structure. The ability to data mine genetics and mutate natural enzymes greatly increases the chemical transformations achievable using biocatalysts.^7,8^ From a life cycle and sustainability point of view, isolated enzyme biocatalysts offer several advantages, including the promise of very high selectivites, high reaction rates under mild operating conditions, and the lack of precious and rare elements in their composition. Nevertheless, protein stability is the Achilles heel of enzyme catalysis, and this can be severely restrictive and may even prevent the adoption of a biocatalytic route. Even if commercialized, many enzymes are only used once before disposal. If an isolated enzyme is used in homogeneous solution, it will suffer from similar separation problems to a homogeneous chemocatalyst, and the protein may not survive the separation process. To maximize the benefits to sustainability that biocatalysts offer, increased biocatalyst stability, improved separations, and increased enzyme recycling are important targets. One approach to achieving these goals is to associate the enzyme with a solid material to immobilize it and render it insoluble.^9,10^

Immobilization using a suitable support medium is an attractive way to increase biocatalyst stability and recyclability.^11−20^ This marriage of biocatalyst and carrier material provides unique opportunities for the creation of synergic combinations. Different enzyme immobilization technologies have been developed including adsorption,^21,22^ covalent attachment,^23−25^ entrapment,^26,27^ and enzyme aggregation.^28,29^ These methods can increase the lifetime of the enzyme and make separations much simpler, saving on energy and solvents. Popular methods in industry include the covalent attachment of the enzyme to a support material, often a functionalized polymer prepared by a third party. For example, polymers that contain epoxy groups can be attacked by nucleophilic groups on amino acids (such as lysine) on the polypeptide of the protein leading to covalent linkage. Popular cross-linking methods such as the formation of cross-linked enzyme aggregates (CLEAs) also require the covalent modification of the protein to cross-link it and render it solid and recyclable. Unfortunately, many enzymes employed in chemical synthesis suffer from short lifetimes under industrial reaction conditions. The reality of operating an isolated enzyme process is that the protein will degrade, denature, and/or become poisoned. Immobilization methods will retard this, but not prevent it, and therefore, sooner or later (and often sooner) the operator is left with a spent enzyme attached to the support material. As chemicals and energy have gone into the production of the support, the disposal of the support and spent enzyme is not ideal. If the enzyme and support are strongly bound together, there may be little other option but to dispose of the support with the enzyme. Therefore, the “greenest” methods of enzyme immobilization will be reversible, so that the full lifetime of the material can be exploited. Many of these will be physical methods without covalent attachment of the protein to the support, such as adsorption and entrapment, but some reversible covalent methods have been reported.^30,31^ The formation of a CLEA also provides a partial solution to this problem, as the amount of added material used (the cross-linker) is relatively low and could be factored into the disposal of the enzyme, for example by ensuring cross-linkers are bioderived and have a lower carbon footprint.

In this study, the aim was to develop a new enzyme immobilization method that is reversible, and such that allows easy separation of the enzyme and support material after use. A physical confinement of the enzyme (entrapment) was chosen, as this can lead to an engineered environment within the material behind a porous matrix, which can protect the protein from poisoning and denaturing. In entrapment the enzyme in solution becomes surrounded by the matrix and is physically immobilized during a polymerization event, leading to a gel. Among the gel materials, silica sol–gel processes are commonly employed for enzyme entrapment.^32−34^ However, a wide variety of other materials can be employed to make gels, including “organic” gelators such as crossed-linked polymers^35^ and low molecular weight gelators (LMWGs).^36^ To design a reversible entrapment, the matrix formation must be reversible; that is, depolymerization should be relatively facile. Reversible gels can be formed when the matrix polymerizes due to the formation of supramolecular, rather than covalent bonds. LMWGs, which form gels due to supramolecular forces, are gaining attention due to their versatile application in drug delivery,^37^ stimuli responsiveness,^38,39^ and waste treatment.^40^ A LMWG forms sufficient intermolecular forces with other LMWG molecules, and the solvent, to form a gel phase. LMWGs can be prepared by chemically modifying amino acids or peptides.^41−43^ Entrapment changes the local environment around the protein by confining the enzyme in the solvent within the matrix. This local environment can be tuned as different liquids and dopants can be coentrapped with the enzyme. The matrix is porous and acts as a selective barrier between the enzyme and the bulk solution. This is quite different from surface immobilization, which does not remove exposure to external environments. Biocatalysts have been entrapped within different gel environments, including hydrogels,^44,45^ organogels,^46,47^ aerogels,^33^ and ionic liquid silica gels.^48^ The method adopted in this study was entrapment within a supramolecular ionic liquid gel.

Entrapment in a gel creates an environment for enzyme activity and avoids the formation of strong irreversible links with the enzyme. Supramolecular gels initiated by LMWGs have the advantage of reversibility and modular molecular design.^49−51^ One of the attractive features of supramolecular gels is the ability to shape into spherical beads, which has been demonstrated for hydrogels using different strategies of forming and stabilizing the spherical shape in polar/nonpolar solvent^52,53^ and in emulsion conditions.^54,55^ Recently, a strategy has been developed to make hybrid hydrogel beads using a combination of a LWMG and a biopolymer (alginate) by a heating–cooling method.^39^ The hybrid hydrogel beads exhibited enhanced thermal and mechanical stability. Hydrogels have applications in catalysis,^56^ antibiotic detection,^57^ and drug delivery.^58,59^ Of the LMWGs, amino acids are attractive (bio)materials as they are natural, biocompatible, readily available, and easily modified. Gelation can be initiated by changes in pH,^60^ temperature,^39^ and light^61^ and in response to enzyme activity,^62^ and LMWGs can be gelled under mild conditions for delicate enzyme immobilization. Enzyme hydrogels (aqueous gels) are known that have exhibited better activity than the corresponding free enzyme.^63,64^ Unfortunately, hydrogels tend to be very susceptible to their environment, swell in high water concentration, and shrink in low water conditions. These changes affect the internal structure of pores and can lead to poor mechanical properties and/or enzyme leaching.

This study is concerned with the entrapment of enzymes within supramolecular ionic liquid gels. It is hoped that the inclusion of ionic liquid (IL) within the gel will improve recycling and long-term stability of the protein. ILs have a very low vapor pressure, and the inclusion of an IL leads to observable improvements in gel behavior. IL gels do not exhibit the same tendencies to dry out, and they are mechanically more robust.^54^

Ionic liquids have been shown to provide a unique and tunable environment for enzymatic activity. ILs comprise cations and anions designed to pack together poorly and therefore remain liquid under standard operating conditions. ILs possess many tunable properties that can be systematically altered such as hydrophilicity, acid–base properties, and intermolecular interactions. The potential of ILs to modify biocatalysts and biocatalytic reactions has been noted many times; some representative literature is referenced.^26,65−68^ ILs can offer enhanced substrate solubility and stability of the enzyme and nonaqueous process development, without the need for organic solvents. ILs have been used in enzyme immobilization methods including sol–gel,^69,70^ IL coating,^71,72^ and IL sponge technology.^66,73^ In these studies, it was shown that IL environments can confer excellent long-term stability to enzymes.

Potential applications of ionic liquid gels include sensor applications. In these applications, the conductivity of the IL can be exploited and the IL polymer gel or “ionogel” used as a solid-state electrolyte, for example, in lactate sensing.^74^ Extending the use of IL gels as solid-state electrolytes to enzyme containing gels, Carvalho et al. gelled 1-ethyl-3-methylimidazolium ethylsulfate containing enzymes cytochrome *c*_3_, hydrogenase, and aldehyde oxidoreductase using gelatin and demonstrated the potential of IL gels containing enzymes in enzymatic fuel cells.^75^ Recently, LMWGs derived from amino acids^42,76^ and carbohydrates^77^ were reported that can form supramolecular gels with a range of ionic liquids, opening the opportunity to entrap enzymes within IL containing supramolecular gels.

**Figure 1:**
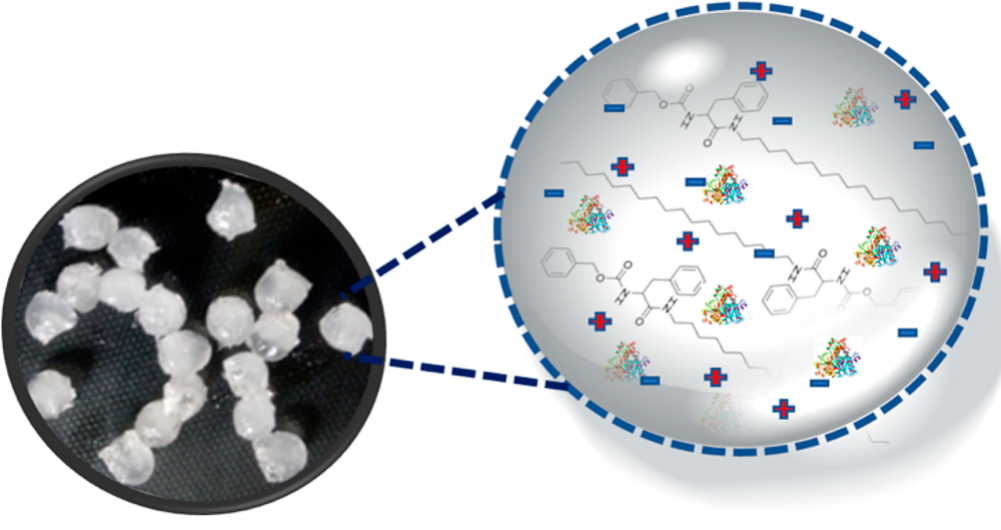
Enzyme containing ionic liquid supramolecular gel beads.

Lipases are interfacial enzymes,^78^ and their industrial applications have been practiced for decades.^79^ As this class of enzyme has so many applications, the harnessing and tuning of lipases from different sources and the development of new lipase immobilization strategies are still active research areas.^80,81^ Due to this prominence, lipases often provide the first test for new immobilization technologies.^17,82−84^ Lipase from *Aneurinibacillus thermoaerophilus* (lipase-AT)^85−88^ is a thermostable enzyme, which has been shown to exhibit hydrolysis activity preferentially for hydrophobic substrates, including substrates of interest in the bioeconomy such as triacyl glycerides olive oil and sunflower oil.^89^ It has been predicted that the interaction of the hydrophobic chain of the substrates and the hydrophobic lid of the enzyme favor the hydrolysis of hydrophobic substrates. Herein, lipase from *Aneurinibacillus thermoaerophilus* (lipase-AT) was entrapped in a LMWG ionic liquid gel to prepare supramolecular gel beads (Figure 1), and the recyclability of the resultant material was demonstrated in the chemical transformation of the model substrate para-nitrophenyl butyrate (pNPB) in aqueous media (Scheme 2).

## Results and Discussions

**Figure 2:**
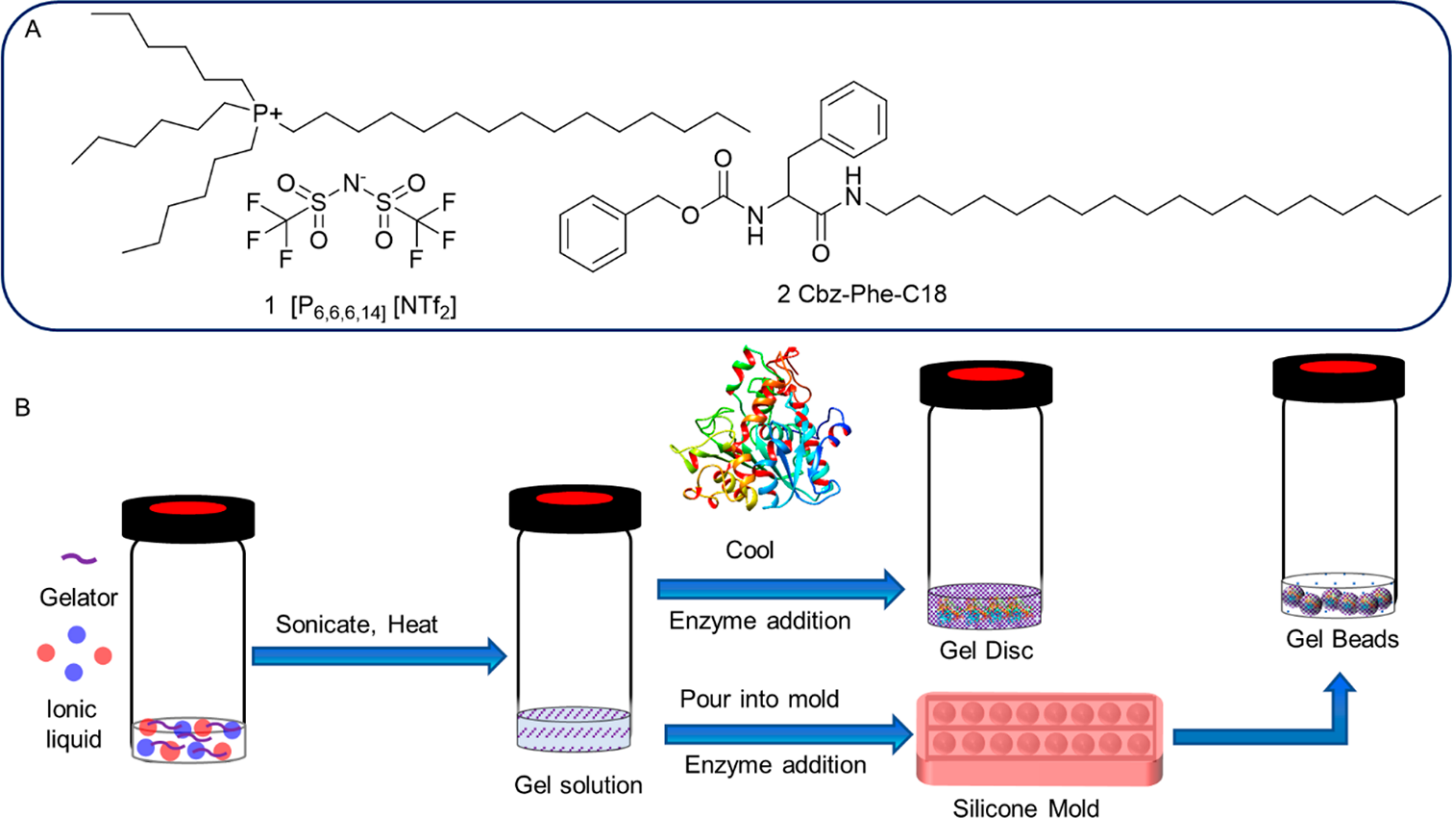
(A) Chemical structure of the ionic liquids [P_6,6,6,14_] [NTf_2_] (**1**) and LMWG Cbz-Phe-C18 (**2**). (B) Schematic of the disc- and bead-shaped lipase-AT immobilized ionic liquid gel formation. Enzyme loading was 1 mg for the gel disc and the gel beads, respectively.

**Scheme 1:**
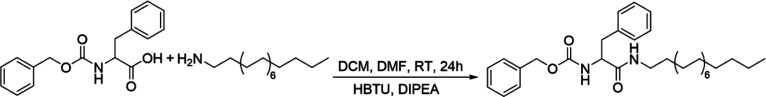
Synthesis of Low Molecular Weight Gelator (LMWG) N-Octadecyl Benzyloxy(carbonyl)-l-phenylalanine (Cbz-Phe-C18)^40^

Hydrophobic ionic liquid (IL) trihexyl tetradecyl phosphonium bis-triflamide ([P_6,6,6,14_][NTf_2_] **1**, Figure 2A), was gelled using LMWG N-octadecyl benzyloxy(carbonyl)-l-phenylalanine (Cbz-Phe-C18, **2**, Figure 2A). LMWG **2** was prepared in one step from benzyloxy(carbonyl)-l-phenylalanine (Scheme 1) according to a literature procedure.^40^**2** was previously used to gel organic solvents and oils. Phosphonium IL **1** and LMWG **2** formed supramolecular gels when heated and cooled. Without the use of a mold, the gel formed as a disc-shaped monolith in the shape of the sample vial (Figure 2B).

The IL and LMWG (0.25, 0.4, 0.5, and 1 wt % with respect to the IL) were mixed in a glass vial and sonicated to form a suspension. The suspension was then heated at 70 °C for 1 h. Upon cooling, a supramolecular gel formed, confirmed by vial inversion. LMWG **2** can gel [P_6,6,6,14_][NTf_2_] (**1**, Figure 2) with super gelation properties at a gelator concentration of 0.50 wt %, giving a transparent gel within 1 h. Increasing the LMWG concentration to 1 wt % reduced the gelation time to <15 min and yielded transparent or translucent gels (Figure S4). The gel–sol transition temperatures of the free and enzyme-doped monolith gels were measured using a heated oil bath and monitored by vial inversion (S.I. Section S4.2.3). The enzyme free gel started to smear at 57.0 ± 1.0 °C, whereas the temperature for lipase-AT added gel was 58.0 ± 1.0 °C. Complete dissolution was observed at 61.0 ± 2 and 63.0 ± 2 °C for enzyme free and lipase-AT added gels, respectively. Successful gel formation requires the right degree of intermolecular forces between LMWG molecules and with the solvent. In this case, LMWG has a high potential for hydrogen bonding (between N–H from the modified amino acid and O=C from the benzyloxycarbonyl group). The high viscosity and nonvolatility of the ionic liquids may also contribute toward good gel formation.

Lipase from *Aneurinibacillus thermoaerophilus* (Almac bioscience) was entrapped in 1 wt % Cbz-Phe-C18 [P_6,6,6,14_][NTf_2_] gels. Lipase-entrapped gel monoliths were prepared by adding and mixing a lipase solution into the gelling solution at temperatures of 37–30 °C during cooling; at this temperature, no immediate gel formation was observed. This avoids the need to heat the enzyme to temperatures that may affect protein structure and function. Opaque enzyme-entrapped gels were obtained within 4–5 min. Typical gel thickness was 2.5–3.0 mm. Full procedures for enzyme entrapment are given in S.I. Section S.4.1. Mechanical properties of the gels were investigated by rheology for the enzyme-doped and undoped materials (S.I. Section S4.2.4). Both materials were found to be viscoelastic.

**Scheme 2:**
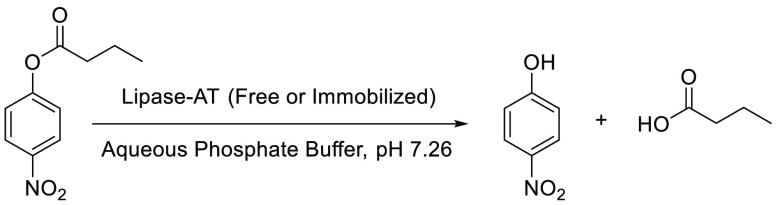
Lipase-AT (Free or Immobilized)-Catalyzed Hydrolysis of Para-Nitrophenyl Butyrate (p-NPB)

The hydrolytic activity of free lipase-AT was investigated using model substrate para-nitrophenyl butyrate (p-NPB) in an aqueous phosphate buffer, followed by UV–visible spectroscopy (Scheme 2, S.I. Section S5.1). The kinetics were investigated by Michaelis–Menten and Hanes–Woolf plots (S.I. Section S5, Figure S10).

For the enzyme immobilization, 1.0 mg (0.1 mL from 10 mg/mL enzyme solution) was entrapped in a monolith. Four parameters of the IL supramolecular gel enzyme entrapment were evaluated: immobilization efficiency, activity, enzyme stability, and recyclability.

During synthesis, the gels were washed with a buffer and the washings monitored by UV–visible spectroscopy (Figure S12). The enzyme loading efficiency of the monolith was determined by measuring the absorbance of three gel wash fractions at 280 nm, and enzyme leaching was quantified by using Bradford assays (Figure S13). In the first wash fraction of the gels, 0–0.01 mg/mL of enzyme was detected by the Bradford assay; however, no detectable enzyme was observed at the second and third wash fractions suggesting >99% enzyme immobilization efficiency in the monolith (Figure S12). A measurable enzyme leaching is expected during the first wash due to unentrapped enzyme adsorbed to the surface of the gels and at the vial walls.

**Figure 3:**
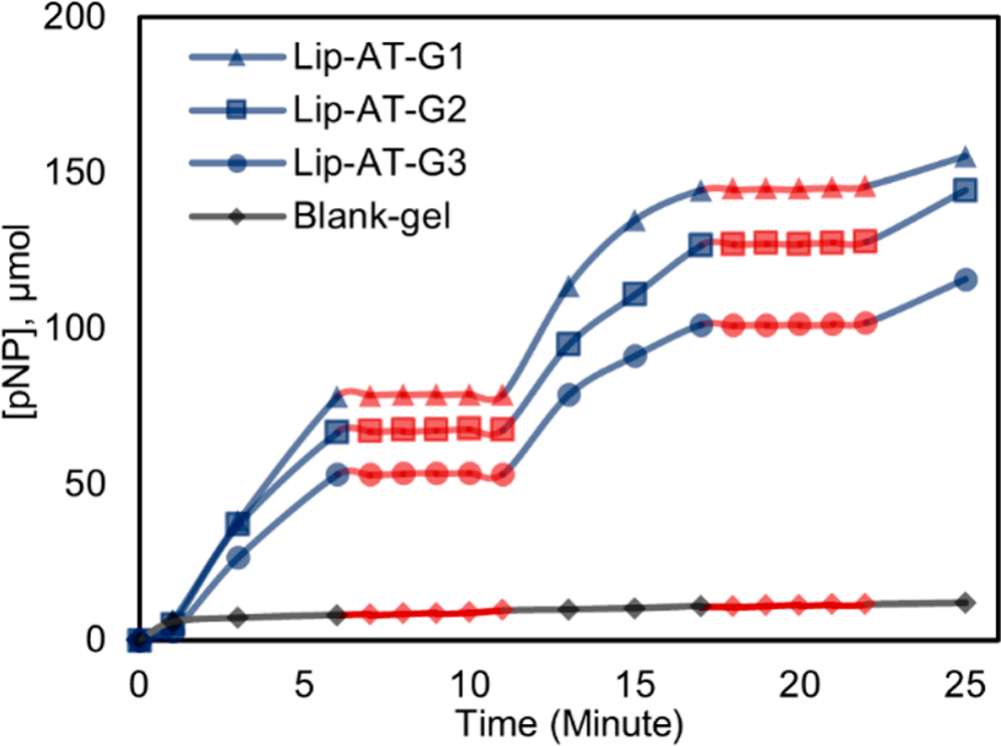
Filtration test of the immobilized lipase-AT in monolith. In the blue regions gel is present; red regions are gel free.

The immobilized lipase-AT in a monolith was active for the hydrolysis of pNPB at room temperature and exhibited a specific activity of 113 ± 21 U/mg of the enzyme. No detectable activity was observed from a blank gel without the entrapped enzyme, suggesting that the activity observed is due to the entrapped enzyme. A filtration test was performed to screen for leaching of active enzyme (Figure 3). The gel was removed from the reaction solution periodically and returned after 5 min. No residual enzyme activity was observed from the assay solution in the absence of the gel, observed as a plateau in the graph, suggesting no leaching of active enzyme was detectable.

The gel monolith was reused over six runs (Figure S14B). There was a reduction in relative activity observed from the first run to the third run. Cycles 3–6 maintained a stable relative activity of 60%–67% initial activity. In the absence of measurable active enzyme leaching, these reductions are proposed to be due to physical changes in the gel, such as pore collapse. Trace amounts of [NTf_2_]^−^ were detected in assay solutions consistent with small structural changes.

**Figure 4:**
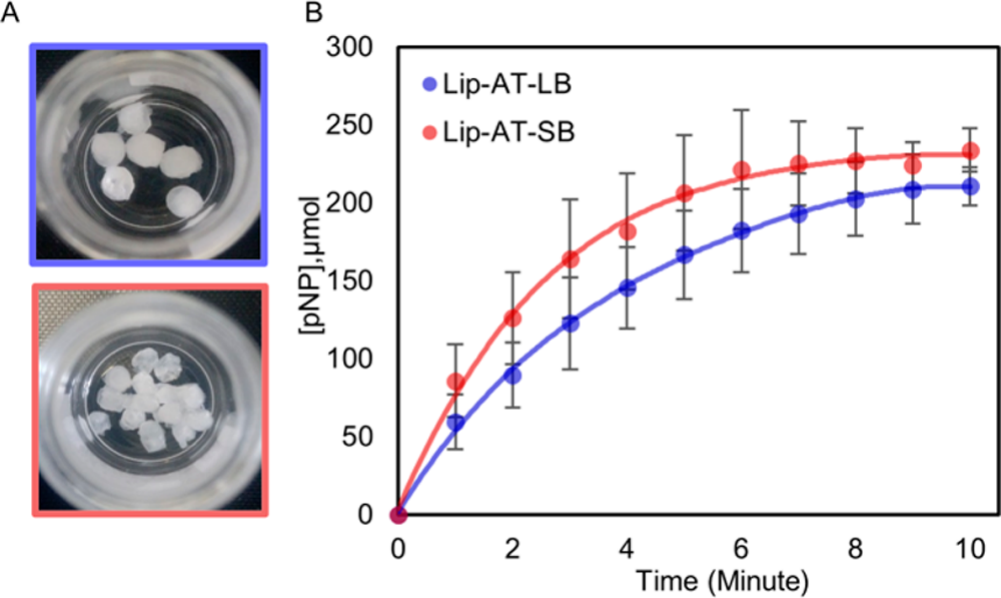
(A) Larger and smaller gel beads. (B) Catalytic activities of larger- and smaller-sized ionic liquid supramolecular gel beads.

The gel was shaped into spherical beads by casting the solution into a silicone mold (Figure 2B). The lipase-entrapped enzyme beads were made by adding the lipase in a buffer into the LMWG/IL solution. The gel was shaped into larger beads with weights of 100.1 ± 8.0 mg/bead and diameters of 3.5–4.0 mm and smaller beads with weights of 45.2 ± 6.0 mg/bead and diameters of 2.7–3.1 mm. The enzyme immobilization efficiencies of the smaller and larger beads were 85% and 96%, respectively. The hydrolytic activity of the gel beads was investigated using pNPB in a phosphate buffer at room temperature (Scheme 2). The numbers of gels beads used were 6 and 14 for the assays of larger and smaller beads, respectively, standardized to contain an equal amount of the ionic liquid, gel, and enzyme. In comparisons to the monolith, the large- and small-sized beads exhibited 5 and 7 times higher activity, respectively. The larger beads exhibited an activity of 569 U/mg enzyme and the small beads 734 U/mg enzyme (Figure 4B). The enhanced activity observed for the smaller beads may be attributable to increased surface area. The small beads produced 14% of the product relative to the free enzyme in 1 min and 21% of the product relative to the free enzyme over 3 min (Figure S18).

It was expected that during the gel preparation small quantities of the enzyme would adhere to the gel surface. The gels were washed three times with high ionic strength buffer solution containing 0.1 M sodium phosphate and 0.15 M NaCl to remove any unbound or weakly surface-bound enzymes form the gel. Filtration tests confirmed that there was no detectable active enzyme in solution, suggesting that there was no unbound or weakly bound active enzyme on the gel surface that could withstand the reaction conditions. Thus, the activity is assumed to be due to enzyme located within the gel. As the environment within the gel is comparatively hydrophobic, it is plausible that the substrate partitions into the ionic liquid phase. It has been shown that hydrophobic ionic liquids can extract polar organic compounds such as diols from aqueous solution.^90^

The hydrolytic activity observed for the gels was lower than the freshly prepared free enzyme solution. This suggests a mass transfer limitation, which is expected and consistent with the observation that as the shape and size of the immobilized enzyme changed from monolith to beads and larger to smaller beads, an increase in activity was observed. The shape should be optimized to enable the most advantageous activity. The specific activity of the enzyme is not the most important parameter in applied pharmaceutical biocatalysis. The overall amount of product that can be prepared and the ease of separation are more important process parameters, as more enzyme can be added to increase rate, and this will be economical if the enzyme can be used many times.

In addition to the shape, the ionic liquid may not be innocent with respect to activity. [P_6,6,6,14_][NTf_2_] is hydrophobic and ionic, and thus, hydrophobic and ionic interactions of the gel with the enzyme may affect activity. Previously it has been shown that [P_6,6,6,14_][NTf_2_] enhances the activity of *Burkholderia cepacia* lipase immobilized in a [P_6,6,6,14_][NTf_2_] ionic liquid silica gel.^91^ A molecular docking study on *Burkholderia cepacia* identified hydrophobic interactions of [P_6,6,6,14_] cations and hydrogen bonding of [NTf_2_] anions with the enzymes, and it was proposed that these contributed to the enhanced activity.^92^ However, high concentrations of hydrophobic ILs reduced *Candida antarctica* lipase B activity.^93^ In this study, the total IL, enzyme, and gel contents of the beads and the monolith were the same; however, as the shape is changed from monolith to larger and then to smaller beads, the amount of gel in contact with the aqueous reaction solution will increase, leading to local changes in the IL:water ratio. The high activity observed for the small beads compared to the large beads may be partly due to a correlation between ionic liquid content and activity.

**Figure 5:**
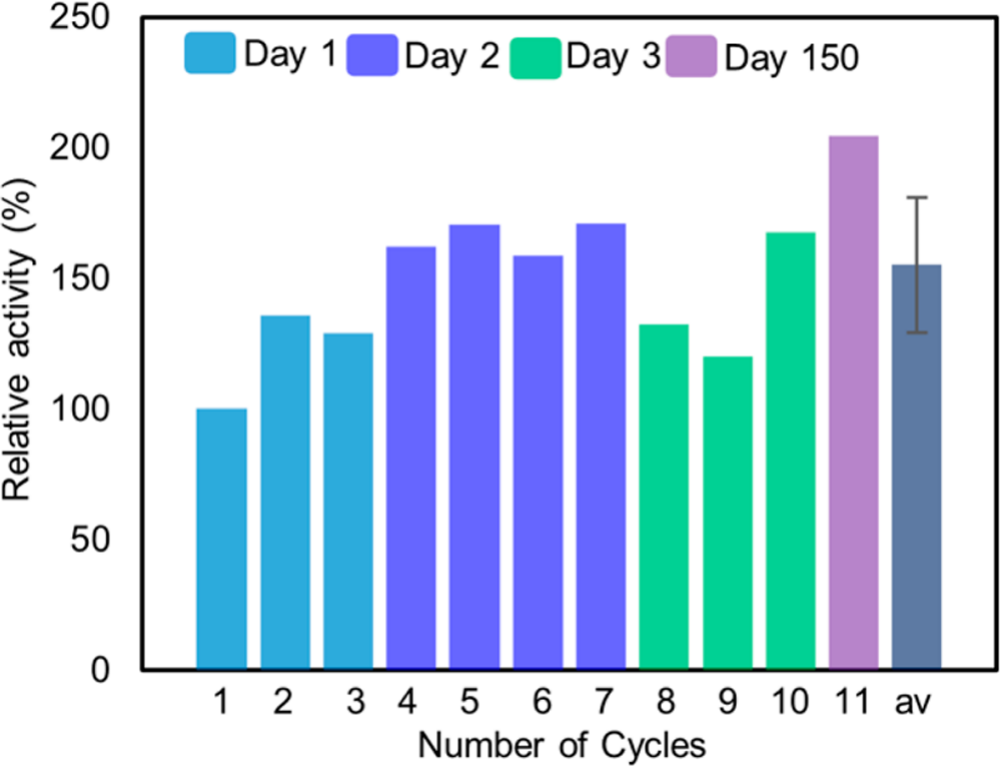
Recycling of ionic liquid supramolecular gel beads (av is the average).

The larger beads were recycled nine times over 3 days without a reduction in activity (Figure 5). To avoid any changes due to drying, the gel beads were then stored in a buffer (0.1 M NaH_2_PO_4_, 0.15 M NaCl, pH 7.26) in a closed glass vial at 4 °C for 150 days. After 150 days, activity was retested and found to be higher than the average activity. In total, 11 catalytic runs were performed. The average activity relative to the first run was 155%.

A filtration test on the 10th and 11th runs revealed no residual activity in the liquid (Figure S16), suggesting that no active enzyme leached from the beads and that the activity observed was due to the entrapped enzyme. The results demonstrated that the supramolecular ionic liquid gel effectively stored the immobilized enzyme for at least 150 days. The increase in relative activity after 150 days is proposed to be due to small changes in the gel structure, which may enhance substrate and product diffusion and/or enzyme activity. In contrast, the activity of free lipase-AT dropped by 23% relative to the free enzyme after 78 days stored at 4 °C (S.I. Section S6, Figure S11).

The smaller beads were recycled for 18 runs over a 147-day period (Figure S17A). The smaller beads showed a greater variation in activity, including a drop-in activity after storage, but remained active over all 18 runs measured. On day 1, higher activity was retained over two recycles. Similar to the large beads, to avoid drying, the gel beads were store in a buffer (0.1 M NaH_2_PO_4_, 0.15 M NaCl, pH 7.26) in a closed glass vial at 4 °C for 143 days. Over days 143 to 147, 15 more recycles were performed. The activity was 59% relative to the first run on the 18th run on day 147. Filtration tests were performed on the 8th, 13th, and 18th runs and suggested that no active enzyme leached from the gels (Figure S17 B–E). Ionic liquid leaching from the gels was investigated. ^19^F NMR spectroscopy was used to monitor [NTf_2_]^−^ anion leaching from the gels to the assay solutions using sodium trifluoroacetate as internal standard (Figure S19). During the storage time for 143 days, a small quantity (0.073 mg/mL) of the [NTf_2_]^−^ anion leached out of the gels as evident from the ^19^F NMR of the gel’s storage buffer (Figure S20A). This was accompanied by a reduction in enzyme gel activity and is consistent with a change to a less favorable structure (e.g., pore collapse). In the subsequent catalytic runs, the activity recovered, markedly peaking on the 11th run. The assay solution from the 4th to 18th runs were investigated for IL leaching; in all but one run, any anion leaching was below the limit of measurement within the spectrum noise. A trace leach of 0.0019 mg/mL of the [NTf_2_]^−^ anion was detected on the 4th run (Figure S20B). The total product formation from 1 mg of free lipase AT was calculated as 808 μmol, giving a turnover number of 3.47 × 10^5^. The corresponding calculation for the small beads containing 0.85 mg of enzyme over 18 cycles gave 21,155 μmol of product and a turnover number of 10.70 × 10^5^.

The small beads exhibited higher activity compared to the larger beads. However, smaller beads had a poorer recyclability and more variation in activity. The larger gel beads exhibited a more regular activity profile from the first use to the 10th use over 3 days, and even after being stored for 150 days, the activity remained similar. These differences could be due to differences in the gel structure, with the smaller beads having a greater surface area and a lower bulk. The gel state is metastable, and therefore, different ratios of matrix:IL and water can produce different local structures and different environments for the enzyme. A small amount of anion leaching was measured during storage and perhaps results in gel microstructure change. However, anion leaching was less pronounced for the large beads, which exhibited a remarkable stability. Mass transport can be faster than expected in ionic liquid gel catalysis, as gels can exhibit remarkable rates of substrate uptake. For example, a molecular Rh catalyst entrapped in an ionic liquid silica gel exhibited higher rates per metal than the homogeneous catalyst and better alkene hydrogenation performance when entrapped in larger, rather than smaller, particle sizes.^94^

The separation of the enzyme and the support was demonstrated by taking an enzyme-doped bead and adding water and ethyl acetate. After agitation on a vortex mixer, the bead dissolved. The upper organic layer was removed, and the volatiles evaporated. Heating and cooling of the resultant liquid led to the formation of a gel revealing the LMWG to be present. The aqueous solution was found to be positive for protein using Bradford reagent. The full procedure and photographs are provided in the S.I. Section S10. This unoptimized procedure demonstrates the potential to partition the LMWG and IL and the protein into different solvents for their separation and the recycling of the LMWG and IL.

## Conclusions and Further Directions

Enzyme entrapment was demonstrated in ionic liquid supramolecular gels using a LMWG based on an amino acid. The procedure is simple, and gelation is reversible. The enzyme can be added at lower temperatures than used for gel initiation. The resultant gels can be shaped or cast, as demonstrated by molding into beads. Supramolecular ionic liquid beads of *Aneurinibacillus thermoaerophilus* lipase (lipase-AT) exhibited good enzyme activity, remarkable stability, long-term storage, and easy recyclability. The immobilized biocatalyst was easily separated from the reaction mixture and recycled. Activity was due to the gel-entrapped enzyme, and enzyme leaching was not detected. The activity measured was dependent on the shape of the matrix, and increasing the surface area increased the relative activity.

Hydrophobic environments protect lipases and help to keep them in an active conformation.^95,96^ By coupling this with entrapment, the enzyme is protected from the bulk solution. The IL used can be altered to suit the enzyme entrapped. In addition, the protein is confined within the pores of the matrix, and this has been shown to reinforce active protein conformations and improve stability.^48,83−85^ Future work will concentrate on the entrapment of different classes of enzymes and using LMWGs and ILs that are bioderived and have greener synthetic routes. The scope of the reactions that can be supported will be investigated. Lipase-AT has a particular preference for natural hydrophobic substrates such as olive oil and sunflower oil,^89^ pointing to potential uses in biodiesel and waste cooking oil upcycling. The IL loading will be varied by altering the IL:water ratio to maximize mass transport and activity, while maintaining advantageous gel physical properties.

One of the big challenges of using LMWG supramolecular gels in catalysis is the control of the shape of the gel due to the weaker (than covalent) bonding that forms the supramolecular network.^50^ Previously, hybrid LMWG–polymer systems have been employed to render the gel robust and shapeable.^39,97^ The supramolecular gels reported here are surprisingly stable, robust, and recyclable, and this reveals the potential for good supramolecular LMWG–IL interactions. The facile shaping of the gel can be exploited to make thin layers and coatings, and this is anticipated to improve mass transport and activity. Coating electrodes will enable use in bioelectrocatalytic applications, due to the ability of an IL gel to act as a solid electrolyte, as demonstrated by Carvalho et al.^75^

Ionic liquid LMWG entrapment processes can be varied by changing the ionic liquid, the enzyme, and the LMWG in a modular fashion. Supramolecular ionic liquid gels are new materials, and as the field progresses, we will understand these materials better, opening up further possibilities for systematic tuning of supramolecular enzyme gels to suit purposes in catalysis, flow chemistry, sensing. and electrochemistry.

## Data Availability

All data supporting this study are provided as Supporting Information accompanying this paper.

## Supporting Information Available

Synthetic procedures, UV/vis, NMR, ATR-FTIR, ESI-MS, elemental analysis, TGA, DSC, Tgel, and rheology. Photographs of gels. Details of catalytic testing procedures, kinetic measurements, filtration tests, recycling, and leach monitoring (PDF)
