# Supplementary material for: Supramolecular Ionic Liquid Gels for Enzyme Entrapment
Source: ACS Sustain Chem Eng. 2023 Apr 24;11(18):6829–37. doi: 10.1021/acssuschemeng.3c00517 (PMC10170508; doi:10.1021/acssuschemeng.3c00517)
Supplement: Supplementary file 1 — sc3c00517_si_001.pdf [file sc3c00517_si_001.pdf]

## Supporting Information

# Supramolecular ionic liquid gels for enzyme entrapment

Hasan T. Imam[a], Kyle Hill[a], Andrew Reid [a], Stefan Mix[b], Patricia C. Marr*[a], Andrew C. Marr*[a]

[a]School of Chemistry and Chemical Engineering, Queen's University Belfast, Belfast, UK.
[b]Almac Bioscience, Almac Group, Almac House, 20 Seagoe Industrial Estate, Craigavon, Belfast, Northern Ireland, UK.

*Corresponding authors: p.marr@qub.ac.uk, a.marr@qub.ac.uk*

**Table of contents**

Lists of Abbreviation S2
S1 Materials and reagents S2
S2 Analytical methods S2
S3 Synthesis and characterization of the gelator and the ionic liquid S2
S3.1 Synthesis of Cbz-Phenylalanine-C18 S2
S3.2 Synthesis and characterization of ionic liquids S4
S4 Preparation and characterization free and enzyme immobilized gel S6
S4.1 Enzyme free (blank) and lipase-AT immobilized gel S6
S4.2 Gel characterization S7
S4.2.1 Thermogravimetric Analyses (TGA) S7
S4.2.2 Differential Scanning Calorimetry (DSC) S8
S4.2.3 Tgel S9
S4.2.4 Rheology S9
S5 Lipase-AT activity and kinetics S10
S5.1 Free Lipase-AT activity S10
S5.2 Free enzyme kinetics S11
S6 Free Lipase-AT storage stability S12
S7 Lipase-AT containing gels: Activity and Recyclability S12
S7.1 Lipase-AT containing gels and enzyme loading S12
S7.2 Activity and recycling of the immobilized enzyme S14
S7.2.1 Data for lipase-AT containing gel monoliths: Activity and Recyclability S15
S7.2.2 Data for lipase-AT containing large and small beads: Activity and Recyclability S16
S8 Comparison of activity of the small beads with equivalent free enzyme S18
S9 Monitoring the ionic liquid leaching from the immobilized gel: $^{19}$F NMR spectroscopy S18
S10 Extraction of the ionic liquid and LMWG from a gel bead S20

Number of pages: 20
Number of tables: 0
Number of figures: 21

**List of Abbreviations**

| | |
|---|---|
| Cbz-Phe-OH | N-Benzyloxycarbonyl-L-Phenylalanine |
| HBTU | Tetramethyl-O-(1H-benzotriazol-1-yl)uronium hexaflurophosphate |
| DIPEA | Diisopropylethylamine |
| pNPB | Para-nitrophenyl butyrate |
| DCM | dichloromethane |
| DMF | Dimethylformamide |
| $[P_{6,6,6,14}][NTf_2]$ | Trihexyl tetradecyl phosphonium bis-triflamide ($[P_{6,6,6,14}][NTf_2]$ |
| Cbz-Phe-C18 | N-Octadecyl benzyloxy(carbonyl)-L- phenylalanine |
| LMWG | Low molecular weight gelator |

## S1 Materials and reagents

N-Benzyloxycarbonyl-L-Phenylalanine (Cbz-Phe-OH), Tetramethyl-O-(1H-benzotriazol-1-yl)uronium hexaflurophosphate (HBTU), Diisopropylethylamine (DIPEA), Octyldecaamine were from TCI, UK. Para-nitrophenyl butyrate (pNPB) and Bradford reagent (for 0.1-1.4 mg/mL protein) from Sigma-Aldrich. Ionic liquids (**S3.2**) were synthesized in-house following standard procedures and verified using published spectra.[1]

All the purchased chemicals from commercial sources were used without further purification.

Lipase from *Aneurinibacillus thermoaerophilus* was donated by Almac bioscience, Craigavon, Belfast, Northern Ireland, UK.

## S2 Analytical methods

NMR spectroscopy: 1D ($^{1}H$, $^{13}C$, $^{19}F$ and $^{31}P$) NMR experiments were performed using Bruker Ultrashield 400 plus spectrometer. At 400 MHz spectrometer, 1D $^{1}H$, 1D $^{13}C$, 1D $^{19}F$ and 1D $^{31}P$ NMR spectra were recorded at operating frequency of 399.91 MHz, 100.56 MHz, 376.44 and 161.96 MHz at spectral width of 20.55 ppm, 238.90 ppm, 237.18 ppm and 51.45 ppm for $^{1}H$, $^{13}C$, $^{19}F$ and $^{31}P$ respectively. With 16 scans for $^{1}H$ and $^{19}F$, and 1024 for $^{13}C$ and 32 for $^{31}P$ NMR.

ATR-FTIR: Infrared (IR) spectra were recorded on an Agilent Cary 630 FTIR spectrometer fitted with an attenuated total reflection (ATR) diamond crystal. The spectra were recorded at 32 scans with a resolution of 8 $cm^{-1}$ at wavenumber ranging from 4000-650 $cm^{-1}$.

Electrospray Time of Flight Mass Spectrometry (ESI-MS) (Waters LCT Premier) and elemental analysis (CHNS) (Perkin Elmer PE2400) were performed using the school facilities service provided by Analytical service and environmental projects division (ASEP), QUB.

Protein quantification, and free/immobilized enzyme activity studies were carried out using Agilent Cary 60 UV-Visible spectrophotometer.

## S3 Synthesis and characterization of the gelator and the ionic liquid

### S3.1 Synthesis of Cbz-Phenylalanine-C18

The low molecular weight gelator (LMWG) was synthesized following a published protocol[2] with little modification. Cbz-Phe-OH (0.6 g, 2 mmol) and 1-Octayldecamine (0.59 g, 2.2 mmol) were dissolved in dichloromethane (50 mL) and DIPEA (0.7 mL, 4 mmol) was added. A solution of

HBTU (0.83g, 2.2 mmol) and DIPEA (0.7 mL, 4 mmol) in DMF (5mL) was slowly added into the Cbz-Phe-OH solution. The mixture was stirred for 24h at room temperature. The solution was diluted with DCM (30 mL) and wash twice with (50 mL) of HCl (1M), $K_2CO_3$ (10% solution), brine and finally with water. The resultant organic phase was dried over anhydrous $MgSO_4$, filtered under gravity and the filtrate evaporated under reduced pressure to give white powder. The product was further purified by dissolving in DCM and crystallize with petroleum ether (40-60%) at 4°C overnight (16 h). A white product was obtained by filtration. The product was dried in a desiccator under vacuum.

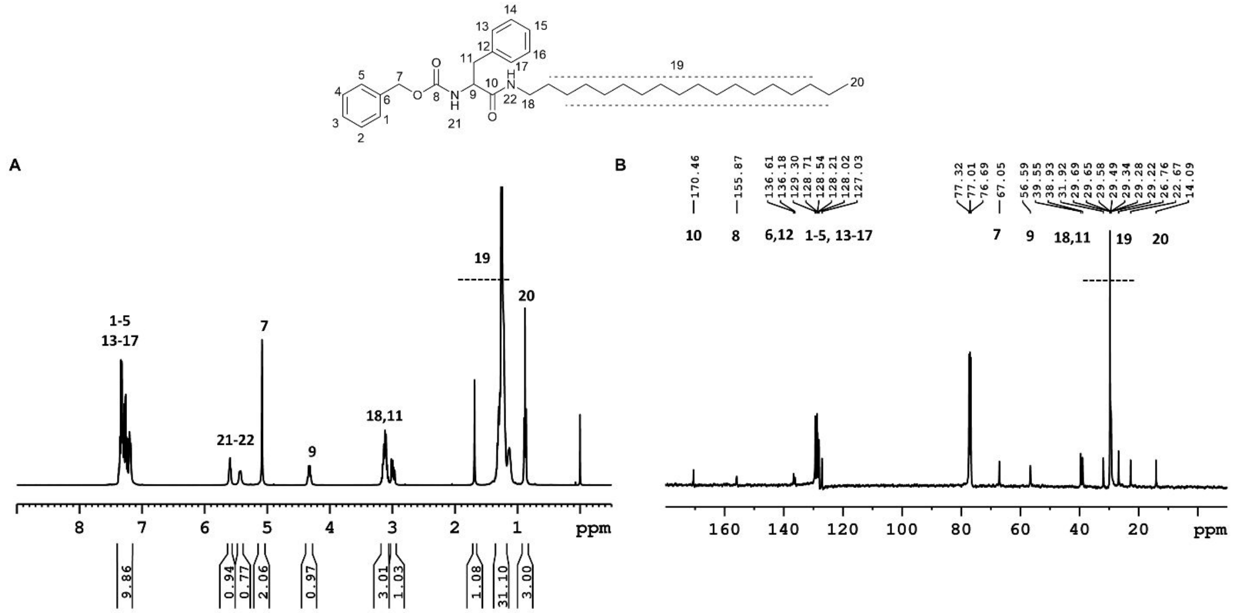

**Fig S1: NMR spectra of the gelator**. A. 1D $^1$H NMR spectrum B. 1D $^{13}$C NMR spectrum

$^1$H NMR (399.91 MHz, $CDCl_3$): $\delta_{(ppm)}$ 7.15–7.39 (m, 10H, **H1-5, H13-17**), 5.59 &5.43 (s, 2H, **NH21-22**) 5.08 (s, 2H, **H7**), 4.33 (t, 1H, **H9**) 3.05-3.18, 2.94-3.02 (m, 4H, **H18**, **H11**), 1.16-1.35,1,68 (m, 32H, **H19**) 0.88 (t, 3H, **H20**).

$^{13}$C NMR (100.56 MHz, $CDCl_3$): $\delta_{(ppm)}$ 170.46(**10**), 155.87(**8**), 136.61(**12**), 136.18(**6**), 129.30(**17**), 128.71(**16**), 128.54(**14**), 128.21(**4**), 128.02(**1**), 127.03(**15**), 67.05(**7**), 56.59(**9**), 39.55(**18**), 38.93(**11**), 31.92, 29.69, 29.65, 29.58, 29.49, 29.34, 29.28, 29.22, 26.76, 22.67 (**19**), 14.09 (**20**).

The obtained $^1$H and $^{13}$C NMR spectra are very similar to that of the published spectra.[2]

ATR-FTIR: Attenuated total reflection- Fourier Transformed Infrared (ATR-FTIR) spectroscopic studies of powder form of the LMWG exhibited characteristics bands for -NH, -CH stretching, amide I, amide II and for carbamate moiety.

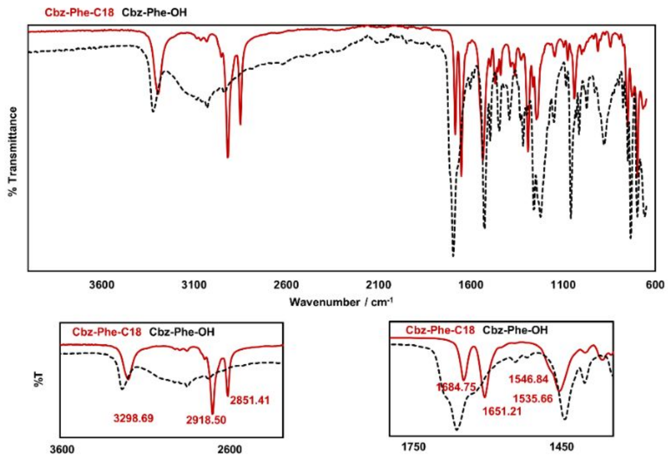


**Fig S2: Overlay of ATR-FTIR spectra** of modified and unmodified LMWG.

In the region of 3300-3200 $cm^{-1}$ the peak observed at 3321 $cm^{-1}$ for Cbz-Phe-OH is assigned to NH stretching, upon alkyl chain (C18) modification, this peak was shifted to 3298 $cm^{-1}$ and two intense peaks appeared at 2918 and 2851 $cm^{-1}$. These are assigned to CH stretching of the alkyl chain. Amide I band (due to C=O stretching) arose at 1651 $cm^{-1}$ and broad band for amide II (due to N-H bending and C-N stretching) was observed at 1546-1535 $cm^{-1}$. A band observed at 1692-1684 $cm^{-1}$ was assigned to the carbamate moiety.

ESI-MS: $C_{35}H_{54}O_3N_2$ calculated 550.8, obtained $[M+H]^+$ m/z 551.4

Elemental analysis: $C_{35}H_{54}O_3N_2$, calculated C 76.32%, H 9.88%, N 5.09% obtained
C 76.03%, H 9.67%, N 5.41%.

**S3.2 Synthesis and characterization of Ionic Liquids**

The ionic liquid was synthesized following the standard procedure[1] with little modification. In a typical reaction, $[P_{6,6,6,14}]$ Cl (30.62g, 0.059 mol) and $Li[NTf_2]$ (20.31 g, 0.071 mol) were dissolved in methanol (40 mL). Deionized water (60 mL) was added into the methanol solution. The mixture was kept stirring for 24 h at room temperature. After methanol evaporation, the product was extracted with dichloromethane (60 mL) and water (60 mL). The organic phase was washed with deionized water (10-20 mL) until no precipitate was observed in a $AgNO_3$ halide test. The IL was dried under vacuum for 16-18 h at 70°C with stirring at 500 rpm.

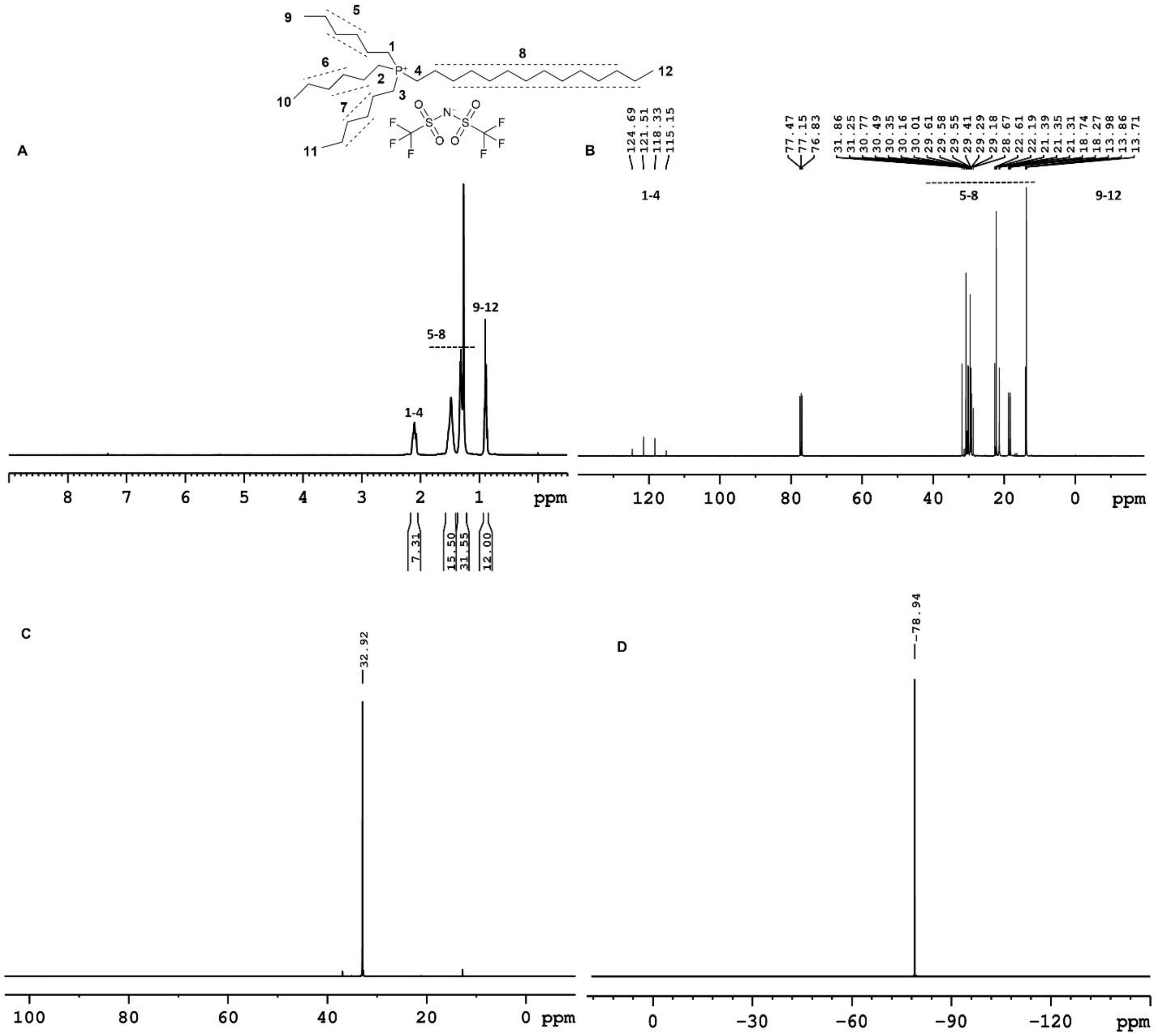


**Fig S3: NMR spectra of the ionic liquid**. A. 1D $^{1}H$ NMR spectrum, B. 1D $^{13}C$ NMR spectrum, C. 1D $^{31}P$ NMR spectrum and D. 1D $^{19}F$ NMR spectrum.

$^{1}H$ NMR (399.91 $CDCl_3$): $\delta_{(ppm)}$ 2.05–2.16 (m, 8H, **H1-4,**), 1.22-1.57 (m, 48H, **H5-8**) 0.85-0.93 (m, 12H, **H9-12**).

$^{13}C$ NMR (100.56 MHz, CDCl3): $\delta_{(ppm)}$ 124.69, 121.51, 118.33, 115.15 (**1-4**), 31.86, 31.25, 30.77, 30.49, 30.35, 30.16, 30.01, 29.61, 29.58, 29.55, 29.41, 29.29, 29.18, 28.67, 22.61, 22.61, 22.19, 21.39, 21.35, 21.31, 18.74, 18.27 (**5-8**), 13.98, 13.86, 13.71(**9-12**).

ESI-MS: $C_{32}H_{68}P$, calculated 483.8 obtained [$M^{+}$]) 483.5. $C_2F_6S_2NO_4$, calculated 280.1, obtained [$M^{-}$] 279.9.

Elemental analysis: $C_{34}H_{68}F_6NO_4PS_2$, calculated C 53.45%, H 8.97%, N 1.83%, obtained C 53.64%, H 9.06%, N 1.85%.

**S4 Preparation and characterization free and enzyme immobilized gel**

**S4.1 Enzyme free (blank) and lipase-AT immobilized gel**

Two different shapes of gel- monolith and beads, were prepared. In a typical gel preparation, LMWG N-Octadecyl benzyloxy(carbonyl) phenylalanine (Cbz-Phe-C18) (typically 3-10 mg, 0.5-1% with respect to ionic liquid weight) was mixed with ionic liquid $[P_{6,6,6,14}][NTf_2]$ by stirring for 5 min in a glass vial (4 mL Screw Neck Vial, 45 x 14.7mm or 14 mL glass vial,14 mL, 48×25 mm) then sonicated for 40 min giving ionic liquid gelator suspension. The gelator suspension was heated in a pre-heated oil-bath at 70ºC for 60 minutes to make a homogeneous gel solution. For the monolith gels, the gel solution was left to cool within the vial at room temperature and the gel formation was confirmed by visual inspection by inverting the vial. Gel beads were made by pipetting the gel solution into spherical shaped silicone molds. 1%wt gels were formed within 10-15 minutes.

The enzyme immobilized gel was achieved similarly. Briefly, lipase-AT (0.1 mL from 10 mg/mL enzyme stock, in 0.1M $H_2NaPO_4$ buffer, pH 7.26) was added to the gel solution (606.5±1.2 mg) in the vial whilst cooling from 37-30ºC (monitored by using Digi-Sense Traceable® IR Laser Gun thermometer) followed by quick shaking to give a disc shaped (monolith) enzyme gel. To prepare gel beads, the gel solution was poured into a silicone mold, then an appropriate amount of lipase was mixed into the gel solution (16.68 µl and 8.34 µl from 10 mg/mL stock, for large and small beads, respectively. 6 of the large beads and 12 of the small beads contained an equivalent amount of enzyme and ionic liquid comparable to the monolith entrapped enzyme.)

In both cases gels were formed within 5-15 minutes. The gels were aged overnight (18-24 h) and washed with assay buffer (2mL × 3 times), first for 16-18 h, then subsequently for 30 minutes twice with shaking at 35 rpm at room temperature. Each of the gel washing fractions were analyzed for enzyme leaching. The buffer washed gels were used for the enzyme activity assay.

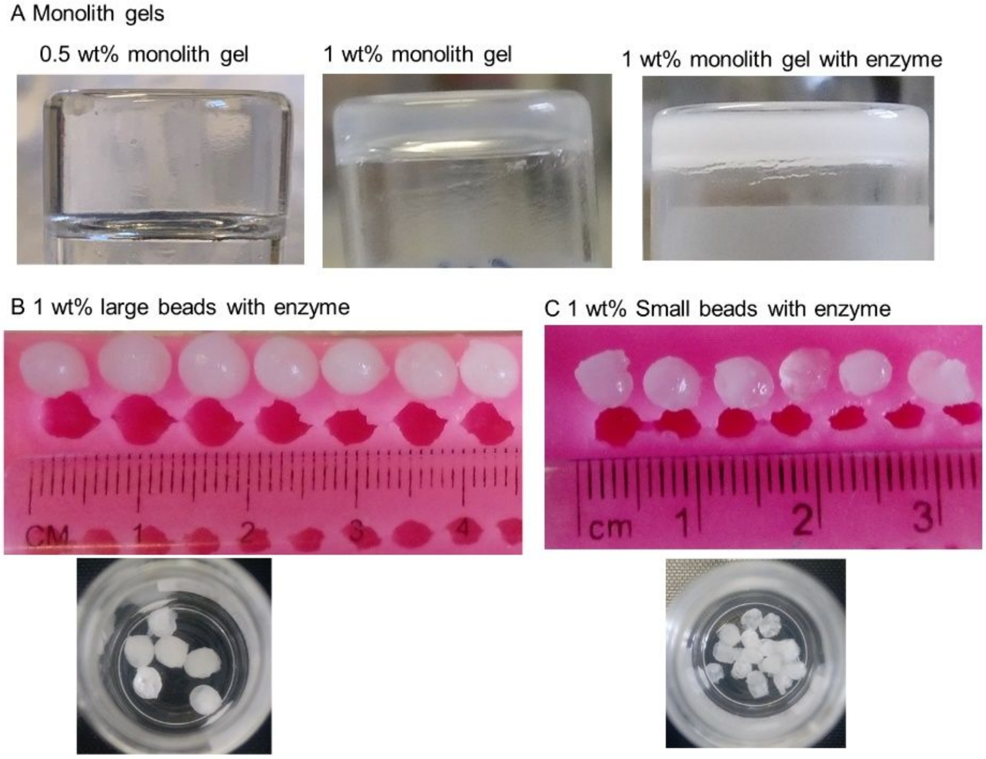


**Fig S4 Three different sized gels**. A. Monolith B. Large beads and C. Small beads.

## S4.2 Gel characterization

### S 4.2.1 Thermogravimetric Analyses (TGA)

Thermogravimetric analyses (TGA) were performed on Thermogravimetric analyzer (TGA 8000, PerkinElmer) instrument under $N_2$ flow at 40 mL/min. In general, 6-10 mg of samples were placed on a TGA pan and heated at temperature range of 25-600ºC at 5ºC/min.

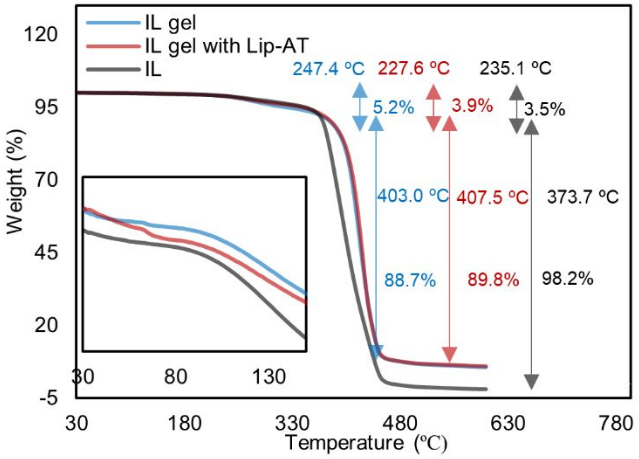


**Fig S5 Thermal properties of the LMWG gel**. Thermogravimetric analysis (TGA) thermogram of IL, IL gel and lipase-AT entrapped IL gel.

Thermogravimetric analysis (TGA) indicated that the thermal decomposition of the gels and the ionic liquid were two steps processes (**Fig S5**). Initially, 3-6% weight loss was observed due to the loss of moisture for all the samples. The enzyme free gel showed higher relative initial decomposition stability compared to lipase-AT added gel and IL, as indicated from the higher initial onset temperature of 247.44ºC for enzyme free gel compared to 227.60ºC for lipase-AT added gel and 235.18ºC for the ionic liquid. At the first decomposition step the onset temperature for the IL was 7.5ºC higher than the lipase-AT added gel. Most of the decomposition occurred at second step with 88.87, 89.84 and 98.23% of free, lipase-AT added gels and IL decomposed. However, in the second step the onset temperature of the lipase-AT added gel was higher of 407.54ºC compared to enzyme free gel 403.02ºC and IL with 373.78 ºC.

**S4.2.2 Differential Scanning Calorimetry (DSC)**

Differential Scanning Calorimetry (DSC) studies were performed using Differential Scanning Calorimeter (DSC 6000, PerkinElmer) under $N_2$ flow at 30 mL/min. Typically, 5-10 mg of sample was heated in a DSC pan and heating cycles were selected as: holding for 5.0 min at 20.0°C and heating from 20.0°C to 80.0°C at 2.00°C/min.

Differential scanning colorimetry (DSC) thermograms of the gel with and without the lipase-AT gave very similar profiles with three peaks (**Fig S6**) within the temperature range of 30-65ºC. The IL does not show any thermal change under the experimental conditions.

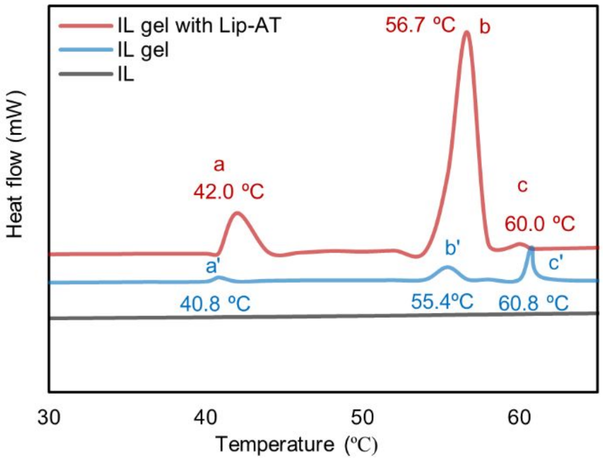


**Fig S6 Thermal properties of the LMWG gel**. Differential scanning colorimetry (DSC) trace of the IL, IL gel and lipase-AT entrapped IL gel.

The two endothermic peaks (**a**, **a'**, **Fig S6)** at 42.00ºC and 40.80ºC, respectively for lipase-AT and enzyme free gels, are due to initial moisture removal from the DSC pan and gel samples. A broad peak appeared at 54.00-58.00ºC (**b, Fig S6**), with a maximum at 56.70ºC for the lipase-AT entrapped gel suggests a gel-to-sol transition, whereas for the enzyme free gel the gel-to-sol transition occurred at 54.00-56.70ºC with a maximum at 55.40ºC (**b', Fig S6**). The broad temperature range for the gel-to-sol transition for the lipase-AT added gel to enzyme free gel suggests that the lipase-AT containing material forms a gel network with thermally distinct properties from the enzyme free gel. The gels exhibited a sharp transition at 60.00 and 60.80ºC for lipase-AT containing and enzyme free gels respectively (**c, c', Fig S6)**, attributed to dissolution of the LMWG.

### S4.2.3 Tgel

The Tgel of the enzyme added and enzyme free gels (0.30 g, with or without lipase-AT, 4mL vial with diameter of 14.7 mm) was measured using a temperature-controlled oil bath **(Fig S7)**. Initially a method was developed with a temperature range of 25-70ºC, at scanning of 5ºC interval. Each temperature was equilibrated for 10 min and monitored by double thermometers, one at the top and one at the bottom of the oil bath. The gels were submerged in the oil bath and maintained at each temperature for 5 min and remove for inspection by vial inversion. Later, the Tgels were measured at temperature range of 55-65ºC and 1ºC interval and each temperature was equilibrated for 5 min. The temperature at which the gel flowed at the edges (smear) was considered as Tgel.

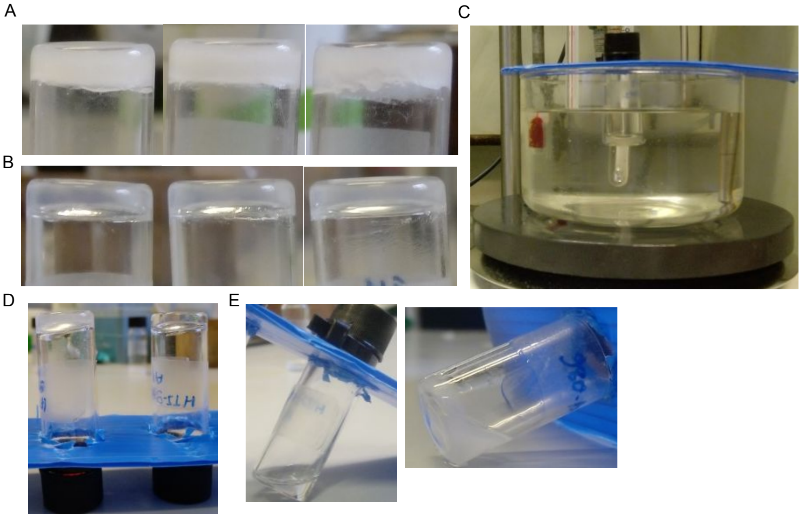


**Fig S7 Tgel determination of the LMWG gel using a temperature-controlled oil bath.** A. Lipase-AT containing gel B. Enzyme free gel C. Temperature-controlled oil bath D. Gels starting to smear at the edge of the vial E. Complete dissolution of the LMWG.

In the oil bath the enzyme free gel started to smear at 57.0 ±1.0ºC, whereas the temperature for lipase-AT added gel was 58.0±1.0 ºC. Complete dissolution was observed at 61.0±2 and 63.0±2 ºC for enzyme free and lipase-AT added gel, respectively.

### S4.2.4 Rheology

The mechanical properties of the gels were investigated using a rheometer R G2 (TA instruments). The frequency sweep experiments were carried out at 100 - 0.1Hz at 25ºC with a controlled %strain variable of 0.5%, using a 40 mm comp HB steel plate (TA instruments).

The mechanical properties storage modulus (G′) and loss modulus (G″) (**Fig S8**) of the gels were investigated using frequency sweep rheology at 0.1-100 Hz, at 25ºC with a controlled strain variable of 0.5%. Both the enzyme free and lipase-AT added gels exhibited higher storage modulus (G′) than the loss modulus (G″) indicating the elastic nature of the gel. The enzyme free gel was mechanically stronger by 1.96 kPa at 0.1 Hz and 5.8 kPa at 79.4 Hz than the lipase-AT added gel as storage modulus (G′) was reduced upon lipase-AT addition. In addition, there are two regions observed for storage modulus (G′), a linear region up to 79.4 Hz after that a bending line is observed with increasing the frequency up to 100 Hz, suggesting some deformation and loss of mechanical strength by a factor of 2.3 kPa and 1.9 kPa for enzyme free and lipase-AT added gel. Similar properties were observed for the loss modulus (G″), but the effect was less pronounced.

The absence of crossover points of the storage modulus (G′) and the loss modulus (G″) suggest an unchanged gel behavior within the experimental frequency sweep. The loss tangent value (tan δ) is an indicator for the elastic and viscoelastic behavior of a material. For elastic materials δ = 0, for viscous materials δ = 90º and for viscoelastic material 0< δ<90º. The initial loss tangent value (tan δ) for both the gels was 0.21 corresponding to a δ value of 11.8º and reached at 0.46 corresponding to 24.7º at 628 rad/s indicative of a viscoelastic nature.

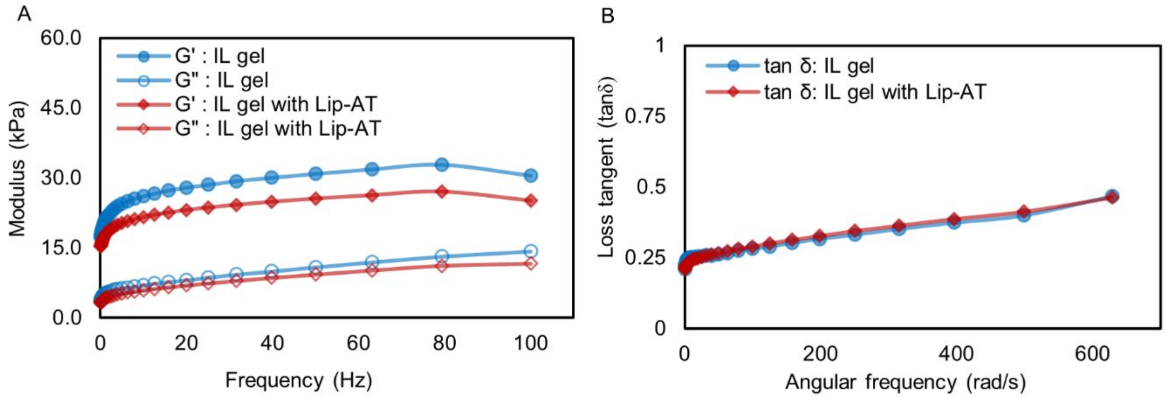


**Fig S8 Rheological properties of the LMWG gel**. A. Loss and storage modulus of the gels. B. Loss of tangent.

## S5 Lipase-AT activity and kinetics.

### S5.1 Free Lipase-AT activity

The hydrolytic activity of lipase from *Aneurinibacillus thermoaerophilus* was investigated by adding the model substrate para-nitrophenyl butyrate (pNPB, in acetonitrile) in aqueous phosphate buffer (0.1M $H_2NaPO_4$, 0.15M NaCl, pH 7.26). The assay conditions were: total volume 2.02 mL, containing 0.005 mg/mL lipase-AT, 0.51 mM pNPB in 1% acetonitrile at room temperature of ~20ºC. The product formation was monitored as a function of absorbance change at 400nm at 1 min/cycle for 10 min by using UV-Visible spectroscopy. Blank containing water instead of lipase-AT under the same experimental conditions was also measured. All the experiments were recorded in triplicate.

The unit of the enzyme has been defied as the amount of the enzyme that produce one micromole (μmol) of para-nitrophenol (pNP) per minute at the experimental condition in aqueous phosphate buffer (0.1M $H_2NaPO_4$, 0.15M NaCl, pH 7.26, containing 1% V/V acetonitrile) using para-nitrophenyl butyrate (pNPB) as substrate. The total enzyme activity has been defined as U/mL and specific activity as U/mg of protein.

Enzyme activity was calculated using the following equations:

Total enzyme activity (U/mL) = $[(\Delta P_{abs}/min - \Delta B_{abs}/min) \times V_R] / [(V_{Lip\text{-}AT} \times E_{PNP})]$ (1)

Specific enzyme activity (U/mg) = A (U/mL)/ Lip-AT (mg/mL) (2)

Where,

$\Delta P_{abs}/min$ = Change in absorbance of the product per minute at 400 nm

$\Delta B_{abs}/min$= Change in absorbance of the blank per minute at 400 nm

$V_R$= Reaction Volume, $V_{Lip\text{-}AT}$ = Lipase-AT volume
$E_{PNP}$ = Extinction co-efficient of pNP at 400nm = 0.016 $\mu M^{-1}cm^{-1}$ obtained from a standard pNP assay Fig S9.

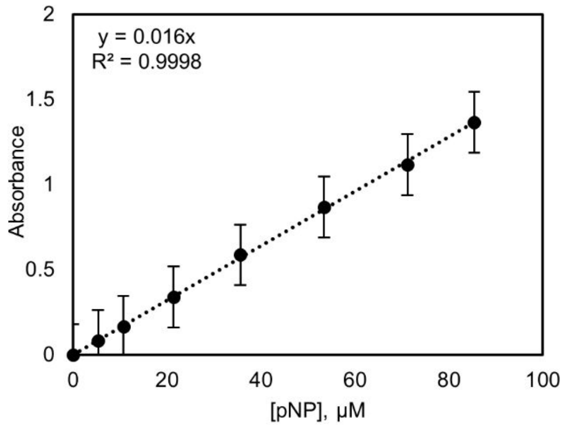


**Fig S9 Para-Nitrophenol (pNP) standard curve**. The molar extinction coefficient of pNP was measured from a standard assay of pNP against sodium hydroxide at the Lipases-AT hydrolytic condition. The assay conditions were: total volume 2.02 mL, aqueous phosphate buffer (0.1M $H_2NaPO_4$, 0.15M NaCl, pH 7.26), sodium hydroxide (0.01M), different concentration of pNP (0, 5.30 ,10.68, 21.40, 35.60, 53.40, 71.20 and 85.40µM) with 1% acetonitrile at room temperature of ~20ºC. The product formation was monitored as a function of absorbance change at 400 nm.

## S5.2 Free enzyme kinetics

The kinetic study of the free lipase-AT was investigated using pNPB as substate in aqueous phosphate buffer (0.1M $H_2NaPO_4$, 0.15M NaCl, pH 7.26). The assay conditions were: total reaction volume 1.01 mL at a fixed lipase-AT concentration of 0.005 mg/mL with different pNPB (in 1% V/V acetonitrile final reaction volume) concentration of 2.04, 1.02, 0.71, 0.51, 0.41, 0.10 and 0.04mM. The product formation was monitored as described in **S5.1** for 5 minutes. Blank containing water instead of lipase-AT was also measured for each concentration of pNPB. All the experiments were recorded in triplicate. Initial rate ($v_0$) at each of the substrate concentrations were plotted against the substrate concentrations [S] in the non-linear curve to fit into the Michaelis-Menten equation to obtain the kinetic parameters Kcat and $K_M$ using solver in-built with excel. The Lip-AT concentration and Kcat were calculated using Lip-AT molecular weight of 43kDa. The kinetic parameters Vmax, Kcat and $K_M$ were also obtained by plotting ratio of the substate to initial rate against substrate concentration ([S]/$v_0$ vs [S]) in the Hanes-Woolf plots.

Michaelis-Menten equation $$v_0 = Vmax[S]/ K_M + [S] \quad (3)$$
Hanes-Woolf equation $$[S]/ v_0 =[S]/Vmax+ K_M/ Vmax \quad (4)$$

Where, $v_0$ is the initial rate, [S] is the substrate concentration.

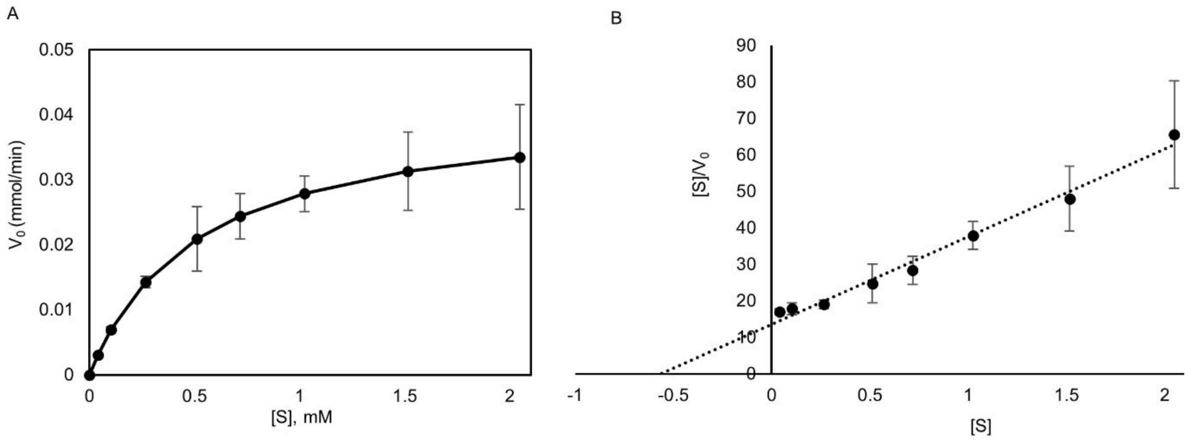


**Fig S10 Investigating the kinetic parameters of free lipase-AT.** A. Michaelis-Menten and B. Hanes-Woolf plots.

The kinetic studies yielded $K_m$ = 0.516±0.050 and 0.556±0.085 mM and $K_{cat}$ of the enzyme was determined to be 6.01±0.15 and 5.88±1.040 $sec^{-1}$ from Michaelis-Menten equation and Hanes-Woolf plots respectively.

## S6 Free Lipase-AT storage stability

An aqueous solution of the free lipase-AT was prepared (10mg/mL, 0.1M $H_2NaPO_4$, pH 7.26) and stored at 4ºC. The activity of the stored lipase-AT was measured as detailed in **S5.1**.

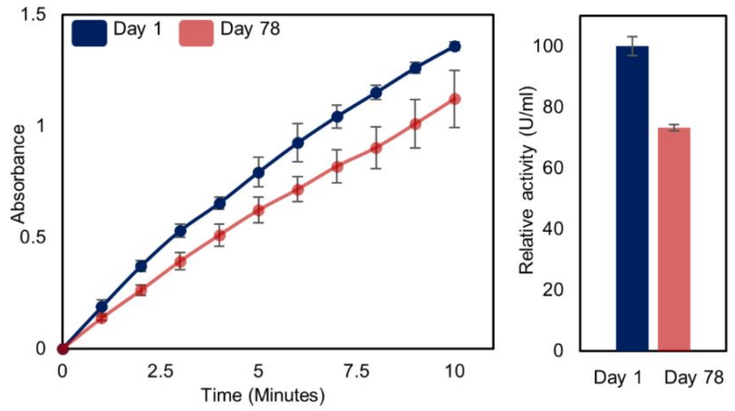


**Fig S11 Storage stability of the free lipase-AT in aqueous phosphate buffer.**

The activity of the free enzyme dropped by 23% relative to the free enzyme after 78 days (**Fig S11**).

## S7 Lipase-AT containing gels: Activity and Recyclability

### S7.1 Lipase-AT containing gels and enzyme loading

Prior to the assay the enzyme containing gels were washed 3 times with buffer (2mL, 1st wash 18h, 2nd and 3rd were 30 min each respectively). The enzyme leaching from the gel to the each of the washing fractions were monitored by using UV-Visible spectroscopy (**Fig S12**) (scan at 600-200 nm, the enzyme has a broad peak at 280 nm due to the presence of aromatic group) coupled with Bradford assays[3] (absorbance measured at 595 nm using the supplier protocol). A standard assay was made for different concentrations of lipase-AT (0.1-1.4 mg/mL by serial dilution in 0.1M $H_2NaPO_4$, 0.15M NaCl, pH 7.26) (**Fig S13**).

The leached enzyme was quantified by Bradford assay and used to calculate enzyme loading efficiency.

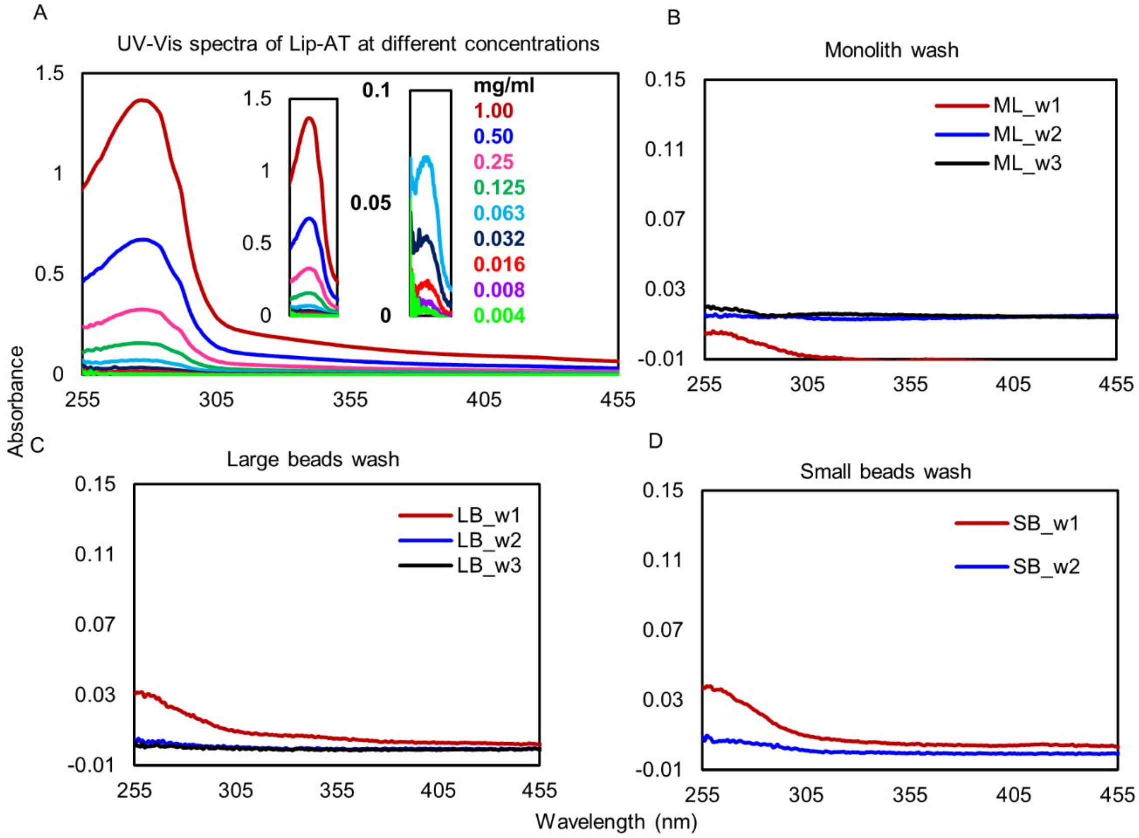


**Fig S12 UV-Visible spectra of free lipase-AT and wash fractions of the enzyme gels**. A. UV-Visible spectra of lipase-AT at different concentration. Washing fractions of B. Monolith C. Large beads and D. Small beads.

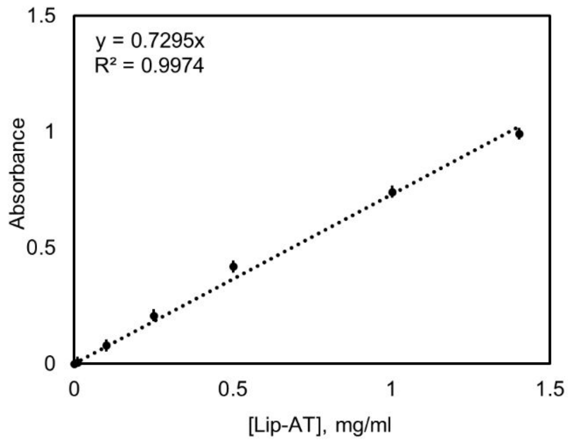


**Fig S13 Lipase-AT standard curve using Bradford reagent**

## S7.2 Activity and recycling of the immobilized enzyme

The hydrolysis activity of immobilized lipase-AT was investigated against para-nitrophenyl butyrate (pNPB) as described in section **S5.1**, however, no additional enzyme was added. Enzyme activity was calculated by measuring the change in absorbance at 400 nm.

For the monolith, the reaction was monitored by taking out the assay solution and returning it to the gel at different time intervals to give assay solution contact times with the gel as 1, 3, 6, 8, 10, 12 and 15 minutes. Between 6 and 8, and 12 and 15 minutes, a filtration assay was performed by measuring the absorbance change of the assay solution for 5 minutes (at 1 min intervals) in the absence of the gel by UV-Visible spectroscopy (600-200 nm). The hydrolysis activity assay was also carried out in the blank gel.

For recycling experiments, the absorbance of the assay solution was measured after 10 minutes.

For the assays of the beads, absorbance spectra (600-200 nm) of the assay solution (25 µl diluted with 275 µl buffer) were measured in one-minute intervals for 10 minutes. The recycling assay was performed by measuring the absorbance of the assay solution after 10 minutes incubation with the gels. The filtration assay was performed between 6 and 8 minutes as described for the monolith. Recycling of the small beads (from the 4$^{th}$ run) were performed using 1% isopropanol instead of 1% acetonitrile.

After each re-use, the gels (monolith and beads) were wash with the assay buffer for 40 minutes with shaking at 35 rpm at room temperature.

### 7.2.1 Data for lipase-AT containing gel monoliths: Activity and Recyclability

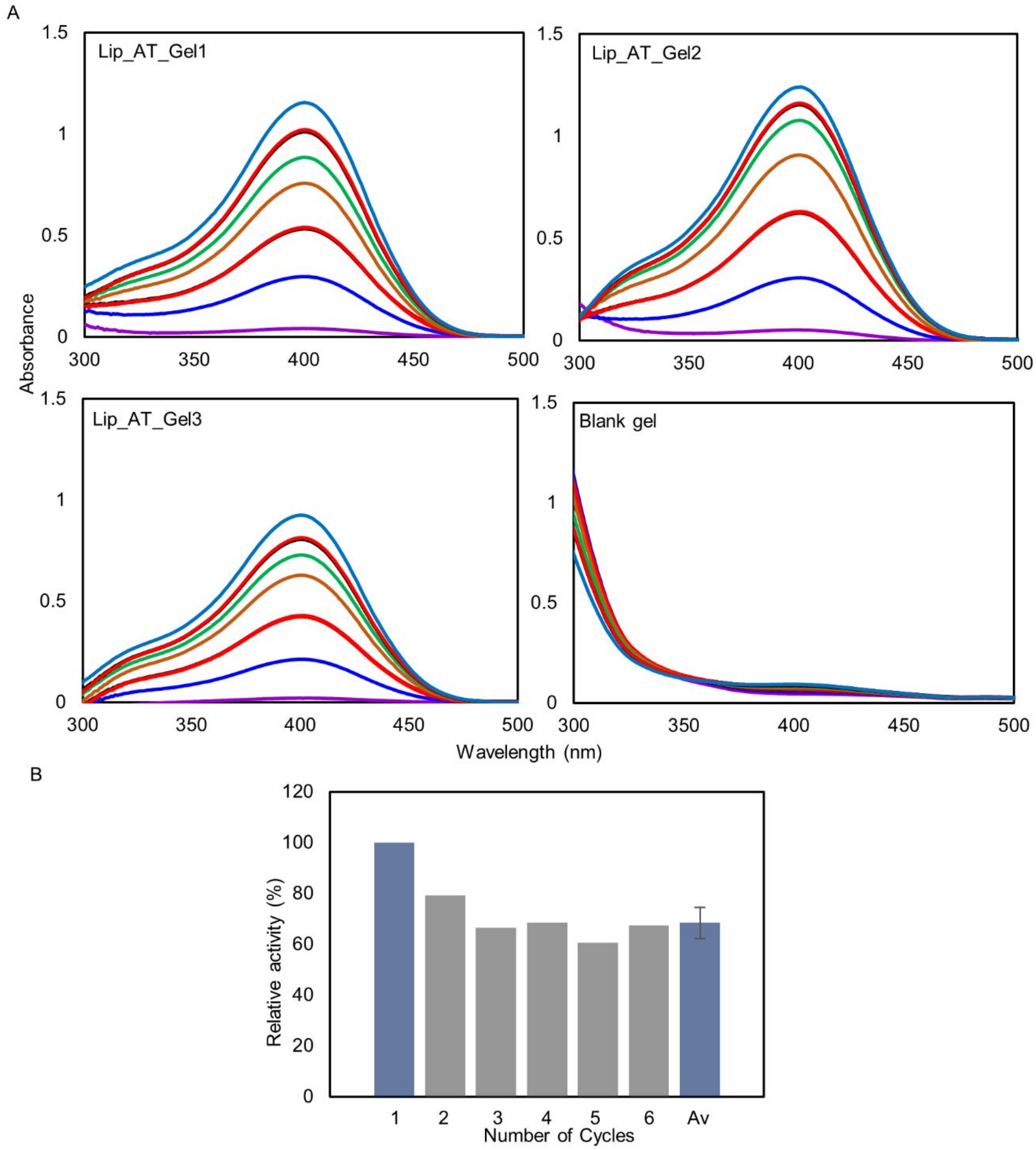


**Fig S14 Activity and recyclability of gel monolith containing lipase-AT.** A. UV-Visible traces of the activity assay, B, Recycling of the monoliths.

## S7.2.2 Data for lipase-AT containing large and small beads: Activity and Recyclability

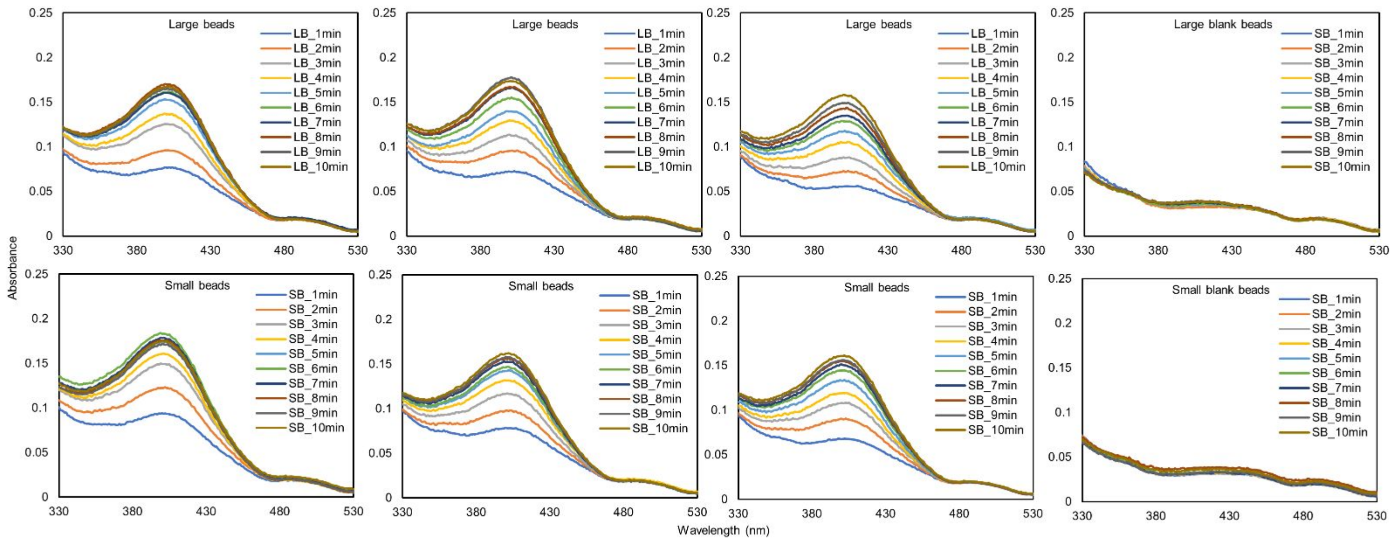

**Fig S15 UV-Visible traces of the activity assays of the large and small beads containing lipase-AT.**

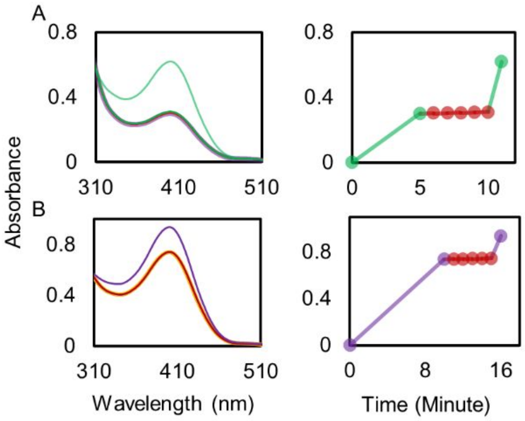

**Fig S16 Filtration assay of the large beads during the recycling assay.** A. Filtration assay of the beads at 10$^{th}$ run at 3$^{rd}$ day. B. Filtration assay of the beads at 11$^{th}$ Run at 150$^{th}$ day.

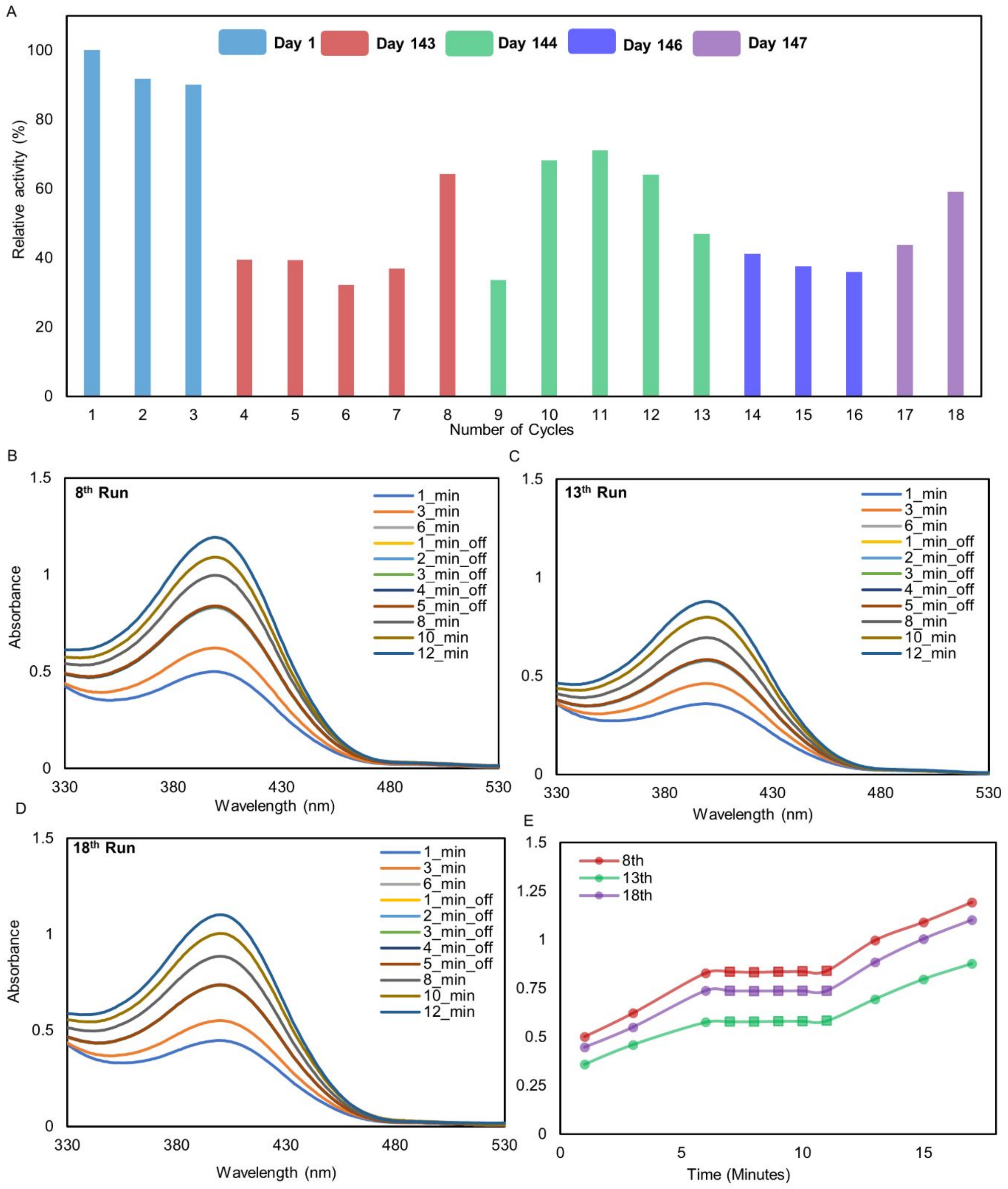


**Fig S17 Recycling, UV-Visible traces and filtration assay of the small beads containing lipase-AT.**

## S8 Comparison of activity of the small beads with equivalent free enzyme

The enzymatic efficiency of small bead immobilized lipase-AT (0.85 mg/mL) was compared to the free enzyme activity of the equivalent amount of free lipase-AT in aqueous phosphate buffer (0.1M $H_2NaPO_4$, 0.15M NaCl, pH 7.26) following the procedures describe in section S5.1.

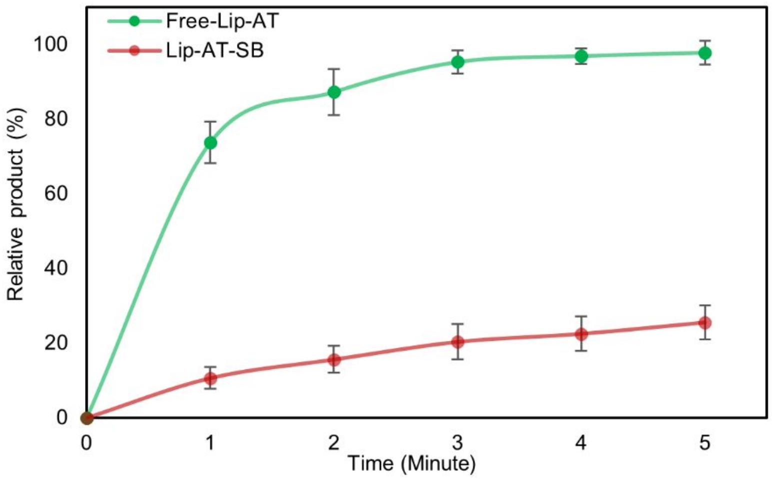


**Fig S18 Comparison of the activity of enzyme immobilized small beads relative to the equivalent amount free lipase-AT.**

## S9 Monitoring the ionic liquid leaching from the immobilized gel: $^{19}$F NMR spectroscopy

Ionic liquid leaching from the immobilized gel was monitored and quantified by using $^{19}$F NMR spectroscopic using Sodium trifluoroacetate as an internal standard. In the enzymatic assay solution (597 µl) sodium triflutoacetate (3 µl from 10.03 mg/mL stock, giving a final concentration of 0.050 mg/mL) was added to give a final volume of 600 µl. The sample was transferred into an NMR tube (Wilmad® NMR tube 5 mm diameter) containing $D_2O$ within a capillary tube. Proton decoupled 1D $^{19}$F NMR spectra were recorded using the parameters described in S2. The amount of the anions leaching was calculated by taking the ratio of the peak's integral of 1 equivalent of fluorine of the [$NTF_2^-$] and the [$CF_3COO^-$], the value was multiplied by the known concentration of [$CF_3COO^-$] in mmol and then converted to mg/mL by multiplying by the molecular weight of the internal standard.

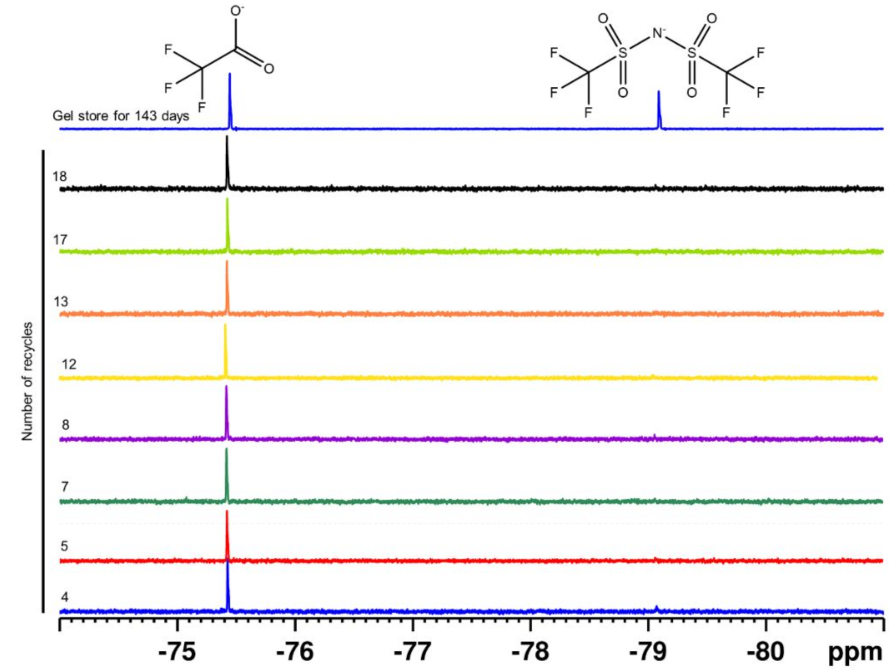


**Fig S19 Monitoring Ionic liquid leaching from the small beads during storage and recycling using $^{19}$F NMR spectroscopy**.

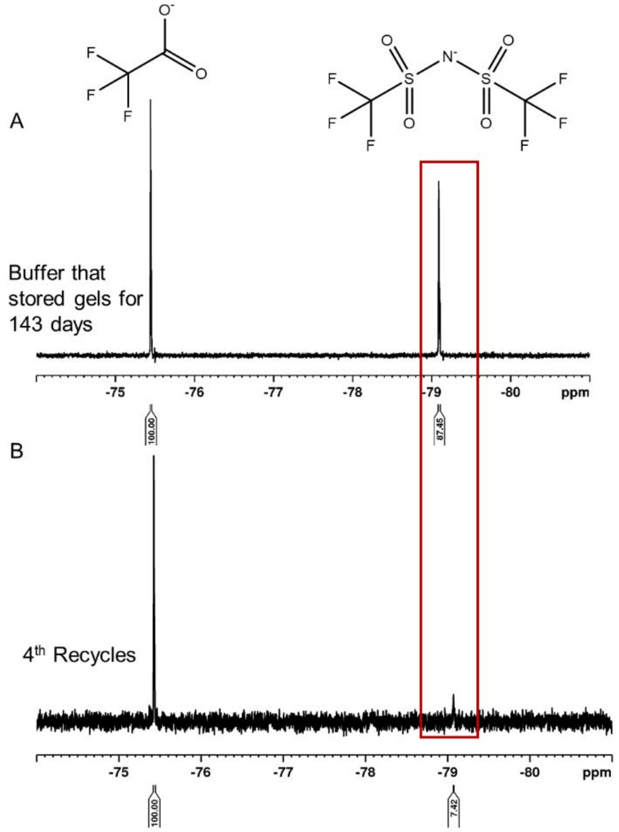


**Fig S20 Detectable ionic liquid leaching from the small beads during storage and the 4th recycle.**

## S10 Extraction of the ionic liquid and LMWG from a gel bead.

One gel bead (larger bead) was removed from storage in buffer and placed in a centrifuge tube (**Fig S21a**). Distilled water (0.5 mL) and ethyl acetate (0.5 mL) were added. The tube was sealed and agitated on a vortex mixer until the bead dissolved. The solutions were centrifuged for 3 minutes at 10000×g (**Fig S21b**). The upper phase was removed, reduced to involatiles on a rotary evaporator then heated in an oil bath at 80ºC for 15 minutes. The resultant transparent liquid was transferred to a mold and formed a gel (**Fig S21c**). The lower phase was tested for protein by adding a drop of Bradford reagent (**Fig S21d**).

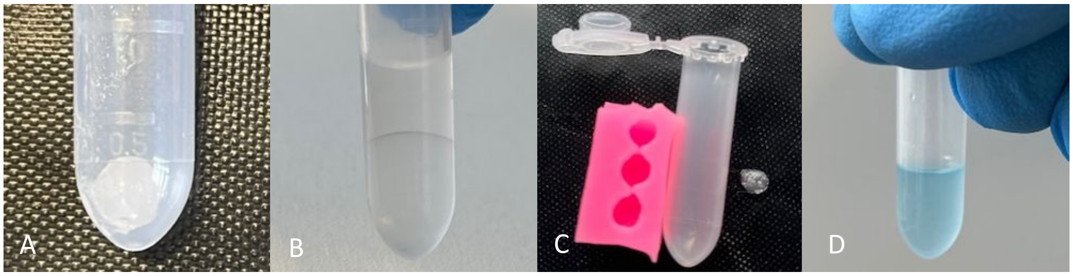


**Fig S21 Stages in the separation of the ionic liquid and LMWG from the protein.** A. A gel bead in a centrifuge tube. B. Aqueous and ethyl acetate layers containing the dissolved bead. C. Mold, centrifuge tube and recovered gel. D. The aqueous layer containing Bradford reagent.

**References**

(1) Grommet, A. B.; Bolliger, J. L.; Browne, C.; Nitschke, J. R. A Triphasic Sorting System: Coordination Cages in Ionic Liquids. *Angew. Chemie* **2015**, *127* (50), 15315–15319. DOI: 10.1002/ange.201505774

(2) Abdellatif, M. M.; Ibrahim, S.; Nomura, K. Efficient and Eco-Friendly Low-Molecular-Weight Gelators Based on L-Phenylalanine as Promising Remediation Tool for Oil Pollution. *J. King Saud Univ. - Sci.* **2020**, *32* (1), 946–951. DOI: 10.1016/j.jksus.2019.06.003

(3) Bradford, M. M. A Rapid and Sensitive Method for the Quantitation of Microgram Quantities of Protein Utilizing the Principle of Protein-Dye Binding. *Anal. Biochem.* **1976**, *72* (1), 248–254. DOI: 10.1016/0003-2697(76)90527-3
